# Supplementary material for: Genome-wide analysis of BpDof genes and the tolerance to drought stress in birch (Betula platyphylla)
Source: PeerJ. 2021 Aug 24;9:e11938. doi: 10.7717/peerj.11938 (PMC8395574; doi:10.7717/peerj.11938)
Supplement: Supplemental Information 1 [file peerj-09-11938-s001.doc]

**Table S1.** The coding sequences and deduced amino acid sequences of *BpDof*.

| Gene | Coding sequences | Deduced amino acid sequences |
| --- | --- | --- |
| *BpDof 1* | ATGATCCAAGAACTGTTGGGAGGTGCAGGCCTTATAGCAGGAGAGAGGAAAATCTCCACCAATAATATTATTGCTTCTTCTTCTCCATCTCCTTCTTCTTCCTCAACAACAACCTCGTCAAATTCAGACAACCAAAACTTGAGGTGTCCGCGATGCGATTCTTCCAACACAAAGTTCTGTTACTACAACAACTACAACCTCACTCAGCCACGTCACTTCTGCAAGACCTGTCGCCGGTATTGGACGAAAGGCGGTGCACTCCGAAACGTTCCGATCGGAGGCGGGTGCCGAAAGAACAAGACTCCTCCCATGTCGGCATCGGTTGGAAAATCATGCTCCGGCAAGATGAAGACCATGTCATCTCATGAGATTGGAAGGTCAGGCGGCGGCCTTGGAAGTACTACTGGGTTTGATCCTGACCTTCCATCAGCTAGCCCGATTCTGTGGGGTTCGCCGCAGAATTCTCATCTCTTGGCTTTGCTGAGAGCTACTCAAAACCCTAACCCTAACCCTAATCACTTGCCTAATTCTCTCAATGTGAAGGAGGAGGGAAATATGGTTGGATCACACATGATGTCGACTGAGCCAACGGCACGGACGTCACTGGGCTTCGATCCTCTAGGCCAGGTCCCTTCCCTGGGCCTGTGTAGCTCTTTCTGGAGAAATAATCAGCTGCAAACGCAACCACAGAATGGCTTTGTAGTTGCTGAAGTTCAAAACAGTGGGATTCAAGAACTGTATCAGCGGCTCAGATCATCATCAACTAATTTCTATGCTGATCACTCACCAATGGTTCTCGGCAACGTGGCTTCTTCAACTTCACCGCCCGCATCCATTTTGGAGTCAACTTCGGTTGCTGGGGTGGAATTGGGGTACTGGAATCCAGCATATTCTTGGTCTGATCTTCCAACTACTAATGGTGCATATCCTTGA | MIQELLGGAGLIAGERKISTNNIIASSSPSPSSSSTTTSSNSDNQNLRCPRCDSSNTKFCYYNNYNLTQPRHFCKTCRRYWTKGGALRNVPIGGGCRKNKTPPMSASVGKSCSGKMKTMSSHEIGRSGGGLGSTTGFDPDLPSASPILWGSPQNSHLLALLRATQNPNPNPNHLPNSLNVKEEGNMVGSHMMSTEPTARTSLGFDPLGQVPSLGLCSSFWRNNQLQTQPQNGFVVAEVQNSGIQELYQRLRSSSTNFYADHSPMVLGNVASSTSPPASILESTSVAGVELGYWNPAYSWSDLPTTNGAYP* |
| *BpDof 2* | ATGCCATCATCTGAGACCGAGGAGGGGAAGGCTGTGAGGGTGCAGCAGAGCCTGGGCCACCCTCACCCGCCGCAGACTCAGCCCTTCCCTTGCCCGCGCTGCGACTCCACCTCCACCAAGTTCTGCTACTACAACAACTACAATCTCTCCCAGCCACGCCACTTCTGCAAGTCCTGCCGCCGTTACTGGACTCAGGGCGGTACCCTCCGCAACGTGCCCTTCGGCGGCGGCACCCGCAAGAACGCGACCGCCAAGCGCACTCGCGCTACCACTTCTTCGTCTTCGTCTTCGTCGTCTTCTTCCTCCACCCTTACCAACGAGGCCGTACCCGCCAACCCCATTCCGGTTTTGCCTCCCGGCGATATGGTCTCGCATGATGTGGGTCTGAATGAGAATGTGGCCGTGGGTGGAGGCTTCAACTTCATGGTGAGCTCGCAGGGTCAGGGGTTCTTGGGCCTGAGCGGGTACGGATTGGGGACCATACCCGGGTTCGACGAGATGGGCGGGTTTGGGTTGGGCAGTAGGCTCTGGACATTCCCTGAAGTCGGTGACTTTGGCGGTGGGAGTGCCATCAATGGTGGTGCTCCGGTGGCTTCTTCAGGGTACGACACGTGGCAGATGGGCGGTGCTGATGGTGGGGATTCCTTTGCTTCCACGGACCTTGCGATTTCAACCCCTGCCCAGGGTTTGGAGTGA | MPSSETEEGKAVRVQQSLGHPHPPQTQPFPCPRCDSTSTKFCYYNNYNLSQPRHFCKSCRRYWTQGGTLRNVPFGGGTRKNATAKRTRATTSSSSSSSSSSSTLTNEAVPANPIPVLPPGDMVSHDVGLNENVAVGGGFNFMVSSQGQGFLGLSGYGLGTIPGFDEMGGFGLGSRLWTFPEVGDFGGGSAINGGAPVASSGYDTWQMGGADGGDSFASTDLAISTPAQGLE* |
| *BpDof 3* | ATGTCGGAAGCGAAAGACCCGGCGATCAAGCTCTTCGGAAAGACGATCCCAGTGCCGGAGATCCCGGCCACCGGGTTGGCGGACTCTACCGGAGCTCCAGCGGCTTCTTCGGGTCCTGTGATTGACGATAGTACCGATCAGGACCATGCTTGTTCGACCAACTCTTCGCCCGAGGCCAACACGGACAGAGATGGAGACGAGCGAGAGGCTGAGAAGGACACATTAGGAGAAAAACCAACTGAGAATAAACAGAAAGATGGAGGCCCACCTGTGTCTTCAGACGAGTTAACGAATCCAGATGCAACCTCAGGAATAAGTGAGAACCCTGCTGAGAAGGATAGTGACAGATTAAAACCTTCGAAGCCCGATGAAGAACAGAGTGAGTCTAGTAATTCACATGAAAAACCTCTGAAGAAACCAGACAAGATACTTCCATGTCCCCGCTGTAATAGCATGGACACCAAGTTCTGTTACTACAACAATTACAACGTCAACCAACCTCGTCATTTCTGCAAGAACTGCCAGAGATACTGGACAGCAGGCGGGACAATGAGGAATGTTCCTGTGGGTGCTGGTCGTCGCAAGAACAAGAATTCTGCTTCTCAGTACCGTCACATAACTGTCTCTGAAGCTCTCCAGAATGCTCGAACTGATCTTCCAAATGGAGTCCACCATCCGTCAATGAAAGCCAACGGCACTGTTCTCACATTTGGCTCGGACGCACCCCTTTGTGAATCAATGGCTTCTGTGTTGAACCTTGCTGATAAAACAGCGCGGAGCTGCACTCGAAATGGATTTCATAATCCTGAAGAACTTAGGATTCCAGTTTCTTGTGGAGGTGAAGAAAATGGGGATGATCGTTCAAATGGAGCTTCAGTCGTAACTTCAAATTCAAGGGATGATCCAGGCGTAACTGCATCACAAGAGCAAGCGATGAGGAATTGTCAAGGCTTCGCGCCTCAACTTCCATGTTTTCCTGGGGCTCCTTGGCCTTATCCATGGAATTCAGCTCAGTGGAGCTCCCCCATATCACCACCTGCTTTCTGCCCTCCAGGCTTTCCTATGCCGTTCTACCCTGCCCCGGCTTATTGGGGTTGTACTGTACCAGGCAATTGGAGCATCCCATGGCTTCCTCAGCCATCATCTCCTAACCATATGGCCCCAAGCTCTGGTCCTAACTCTCCAACCTTGGGGAAACACTCAAGAGATGAGAACATTCTCAAATCAAGCAACTATGGGGTTGAAGAACCACCAAAGGAAAACAATGCTGCCGAGAGATGCCTTTGGATTCCAAAGACATTGAGAATTGATGACCCAGGAGAAGCTGCAAGAAGCTCTATATGGGAAACACTGGGGATTAAGAATGACAAGCCTGACTCGATTAGTGGGGAAGGGCTATTTAAGGCATTCCAATCAAAGACTGTTGAAAAGAATAACATAGCTGAAACCTCACCAGTCTTGCAAGCCAATCCGGCAGCATTGTCGAGACAGATTGGCGATGTTCAACTGGTTCATTCAAATTGCCATCAACCGTGCATGCCATGGGATCATTTTGAGTTCGCCAGCCTCATTTCTCAGAATCAGTTACCAGTCCAACCGAGAAGAGCAGAGGAGGATTATCCCGCCATCTCCTCTTTAGGAGAAGATAGCGACTTTAGTTTAGCCATAGGAGCTGATTTCATGAGTTAG | MSEAKDPAIKLFGKTIPVPEIPATGLADSTGAPAASSGPVIDDSTDQDHACSTNSSPEANTDRDGDEREAEKDTLGEKPTENKQKDGGPPVSSDELTNPDATSGISENPAEKDSDRLKPSKPDEEQSESSNSHEKPLKKPDKILPCPRCNSMDTKFCYYNNYNVNQPRHFCKNCQRYWTAGGTMRNVPVGAGRRKNKNSASQYRHITVSEALQNARTDLPNGVHHPSMKANGTVLTFGSDAPLCESMASVLNLADKTARSCTRNGFHNPEELRIPVSCGGEENGDDRSNGASVVTSNSRDDPGVTASQEQAMRNCQGFAPQLPCFPGAPWPYPWNSAQWSSPISPPAFCPPGFPMPFYPAPAYWGCTVPGNWSIPWLPQPSSPNHMAPSSGPNSPTLGKHSRDENILKSSNYGVEEPPKENNAAERCLWIPKTLRIDDPGEAARSSIWETLGIKNDKPDSISGEGLFKAFQSKTVEKNNIAETSPVLQANPAALSRQIGDVQLVHSNCHQPCMPWDHFEFASLISQNQLPVQPRRAEEDYPAISSLGEDSDFSLAIGADFMS* |
| *BpDof 4* | ATGTTTGGTCAGCAAACGTTGCAGTGCCCTCCTCGGCCATTGCCGATGGACAGAAGCTGGAAACCCAACGTTGAAATCGCTCCCAACTGCCCTCGTTGCGCCTCTTCCAACACAAAATTCTGTTACTACAACAACTACAGCTTGTCACAGCCTAGGTACTTCTGCAAAGGCTGTCGGAGGTACTGGACCAAAGGCGGGTCTCTTAGAAACGTACCTGTCGGCGGCGGTTGTCGGAAAACTCGCCGTGCCAAGTCGGCTAGGCGGGTTTCGCTAACTTCTTCTCATAATGCCAATGATCAGCAATCAACTGACTCTTGTTCCTCTAATGGGGACTCCGTCTCTCAACGAAGCGAAACCAACGGGTCGGATATCGATCTTGCGGTTGTTTTCTCCAGGTTCTTGAATCAGAATCCAAGCAATATTGAGCCGGCGTTCACAGCAGTCCCGGAATTTCCTAATAATTCCAACGCACCGTCCGATTCGCCCAAGTCTTCGAATCCGGAATTTCCCAATATACTGGAAGGACTTTCTGGAGAAGAAAAAGTTGGAGAATTCATTGAAGACGATGTGAATGCATTCGCGTTGGAGGCTTTGTTGAATGATGAAGTGGACCAAGATGTTTTGTGGTCCGATGCTGCAACTTTGCCGAATTTAATGTGGCAACCTATGATGCAATTGCAAGAGTTGGAGTCGCCGTTTCAGTCCAATAATCAGCTAAGGAATTCTACGAATCTAATAACCGACGGTTGGAGTTCCTTGGATCTGTCAGGTTTCGAGGTTTTTTCAAGACCTTAA | MFGQQTLQCPPRPLPMDRSWKPNVEIAPNCPRCASSNTKFCYYNNYSLSQPRYFCKGCRRYWTKGGSLRNVPVGGGCRKTRRAKSARRVSLTSSHNANDQQSTDSCSSNGDSVSQRSETNGSDIDLAVVFSRFLNQNPSNIEPAFTAVPEFPNNSNAPSDSPKSSNPEFPNILEGLSGEEKVGEFIEDDVNAFALEALLNDEVDQDVLWSDAATLPNLMWQPMMQLQELESPFQSNNQLRNSTNLITDGWSSLDLSGFEVFSRP* |
| *BpDof 5* | ATGGAAATGGAAAGAGAATGGAAACCAAACGTTGACATATCCCAAAATTGCCCTCGATGTGGTTCTTGCAACACAAAATTCTGCTACTACAACAACTACAGCTTGACACAACCCAGGTACTTTTGCAAGGGCTGTAGAAGGTATTGGACTAAAGGCGGGTCCCTCCGGAACGTCCCCGTCGGCGGCGGCTGCCGGAAAAACAGACGGGGCAACAAGTCCTTAAGGCTATCCAGTAGTACTGAGGGTCATTTGGCCCATGGGAAGCTCCCAAATGACCCAATGGGCCGCCATCATTCCTTGGTTGGGCCATCAGCCTCCGGCTCCACAATGATGCCTGATGGTTCACACATTGATCTTGCACTTGTATATGCAAACTTCTTGAATCAAAAGCCAGAGTCGAAGACGTCGCCGTTTGAGTTGCCAGAATTGCCTGCTGATCATTGTTTCGTGAGCTCAAGTATTTCTCCATTATCAGTGGAAGAAAATGGTTTTTTTGGATGCCACACTACTCTTTCAGGTGAAGAATCTGTAAGGTATGACGACTCAAGCAATTATTATGCGTTGCCACCATTGCCTGGAGAAGAAGTAGTAGTGTGGTCGAGTTCTCAAGCAATGATGGTGAATCATAGCTTTCAAGGAACACAGCTGCCTGCAGTGCTCGGACCAGAAGCAGAAGAATATCCGGATTCATTGATTTCTAATTGGAGCCCGTTCGATACTTTCTCAAGGACATGA | MEMEREWKPNVDISQNCPRCGSCNTKFCYYNNYSLTQPRYFCKGCRRYWTKGGSLRNVPVGGGCRKNRRGNKSLRLSSSTEGHLAHGKLPNDPMGRHHSLVGPSASGSTMMPDGSHIDLALVYANFLNQKPESKTSPFELPELPADHCFVSSSISPLSVEENGFFGCHTTLSGEESVRYDDSSNYYALPPLPGEEVVVWSSSQAMMVNHSFQGTQLPAVLGPEAEEYPDSLISNWSPFDTFSRT* |
| *BpDof 6* | ATGGTTTTCTCATCCGTTCCCGTCTATCTGGATCCACCCAACTGGCAACACCAGCAACAAAATTATCAACAACCACCACCAGGTGGCCATGGAAGTCATGAGAATCCCCAGCTCCCACCTCCGCCGCCGCCACCTGTTGGAAGCGGAGGTGGCACCACAGGGTCGATCAGGCCAGGTTCGATGGCGGATCGAGCCCGGCAAGCGAAGATTCCACAGCCGGAGACGACCCTGAAATGCCCGAGATGCGAATCCACAAACACAAAGTTTTGCTACTTCAACAACTATAGCCTTACTCAGCCCCGTCACTTTTGCAAGACCTGTCGGCGATATTGGACAAGAGGAGGTGCACTAAGGAACGTGCCGGTTGGCGGTGGTTGTAGAAGAAACAAAAGAAGCAAAAGCAGTAGTAGATCGAAATCTCCGGCCACCGGCGGCGAGCGCCAACCTGGGTCTAGTAGTACTTCTGTTTCTTCCGCTGATATGTTAGGCCATTTCCCCCCTCCACCACCACCAAACTTGTCATTTTTGCCTCCTTTGCATCACCTCAGTGAATATGCTTCCGGGGACATTGGGTTAAATTTCGGCGGATTTCAGCCTCCTGTGGCGGCAACAGGCGGTGATGTTCAGTTTCAAACTGGTGGTAGGTCTTATTTATCAACTGGGTTTGCTGAGCAGTGGAGAATGCAACAAGTGCAGCAGTTTCCTTTCTTGGCAAATTTGGAACAACCAATTGGGTTGTTTCAATATGATGATAATCAGGGTGAGAATGTTGAGGCGACGAACTATGTAGGGGTAGCTGGGCAGCTCCGGTCTAAGCCGTTGGATTCAGAAATATCAGTGAAAATGGAAGAAAATCAAGGGCTGAATTTGTCAAGATCTCATTTGGGTAGTACTTCAGGAAATGATCAATACTCATGGGGTGGTGCCAATGGCAATGGCAATGGCAATATTGCATGGACTGATTTTTCTGCTTTCACCTGA | YFNNYSLTQPRHFCKTCRRYWTRGGALRNVPVGGGCRRNKRSKSSSRSKSPATGGERQPGSSSTSVSSADMLGHFPPPPPPNLSFLPPLHHLSEYASGDIGLNFGGFQPPVAATGGDVQFQTGGRSYLSTGFAEQWRMQQVQQFPFLANLEQPIGLFQYDDNQGENVEATNYVGVAGQLRSKPLDSEISVKMEENQGLNLSRSHLGSTSGNDQYSWGGANGNGNGNIAWTDFSAFT* |
| *BpDof 7* | ATGGTTTTTTCTTCCATCCCAGCTTATCTTGATCCAGCCAACTGGCAACAACAGCAGCCAAATCATCCAACTGGGACTAGTATTGCAGGGAGTTCGCAGCATCTTCCACCTCCTCCGCCCCCGCCGCCACCCCCGCATCCTCATGGGGCCGGCAGCGTGGGCTCGATCAGGCCCGGGTCAATGGCGGATCGAGCTCGGATGGCGAACATACCGTTGCCGGAGGCCGCGCTAAAATGCCCAAGATGCGACTCCACAAACACTAAATTTTGCTACTTCAACAACTACAGCCTCACCCAGCCTCGCCACTTCTGCAAGACCTGCAGAAGGTACTGGACTAGAGGCGGCGCTCTTAGAAATGTCCCCGTCGGAGGAGGCTGTCGGAGGAACAAAAGGAGCAAAGGGAGTAGTTCAAAGTCTCCGGCGAGCAGCGCCGATCGCCAATCGGGTAGTGCTGGTTCCGCCAGCTCAATTCCTTCTAATAGTGGCACCGCCGATATGATAGGCCATGGCCTCAACCCCCAGCTACGATTCATGGCTCCTTTGCATCAACTCTCTCATGACTTTGCTGCCGGGGAGATGGGCTTGAACTATGCCTTGAATTATACACCAATGGGAGTAGCTCCTGGTGACTTGAATTTTCAGATAGCAAGTGGTGGTGGTGGTGGCGGCGGTGGCGGTGATGCTGGGTCCTTTTTATCGGAGCACTGGCGGTTGCAGCAAGCCCAGGCGGCCCAGTTTCCGTTCATGGGTGGGTTGGATCCTTCGCCGGGCGGGTTGTTTGGAGGCAGTGTTGAGGCATCGGGTTATCTAGGGGGAACGAGTCACGGCCGAAGTACCCGGCCGTCTAGCTCGGGGGTTACCCAGCAGATGGCTTCAGTGAAAATGGAGGACAATCAGGAGCTGAATTTGTCGAGACAGTTGTTGGGAATTCCTGGGAGTGAGCAATACTGGACTACTGGTAATACTGCATGGACAGATCTTTCTGCTTTTAGCTCTTCTTCCACTACAAGGAACCCCCTATAG | MVFSSIPAYLDPANWQQQQPNHPTGTSIAGSSQHLPPPPPPPPPPHPHGAGSVGSIRPGSMADRARMANIPLPEAALKCPRCDSTNTKFCYFNNYSLTQPRHFCKTCRRYWTRGGALRNVPVGGGCRRNKRSKGSSSKSPASSADRQSGSAGSASSIPSNSGTADMIGHGLNPQLRFMAPLHQLSHDFAAGEMGLNYALNYTPMGVAPGDLNFQIASGGGGGGGGGDAGSFLSEHWRLQQAQAAQFPFMGGLDPSPGGLFGGSVEASGYLGGTSHGRSTRPSSSGVTQQMASVKMEDNQELNLSRQLLGIPGSEQYWTTGNTAWTDLSAFSSSSTTRNPL* |
| *BpDof 8* | ATGCAAGACATGCATTCGATCGGAGGCAGCGGGCGGTTATTCAGCGGCGGCGCAGGTGGAGGAGGAGGGGACCGGAGGGTTCGACCGCACCACAACCAGCACCACCAGCACAACCACCAGGCGCTCAAGTGCCCGCGGTGCGACTCGCTCAACACCAAGTTCTGCTACTACAATAACTACAACCTATCTCAGCCCCGCCACTTCTGCAAGAGCTGCCGCCGCTACTGGACCAAAGGTGGCGTCCTCCGCAACGTCCCAGTCGGCGGCGGCTGCCGGAAGGCGAAGCGCTCCAAGACTAAGCCCTCCTCCGACGCATCAGCGCCACCGCCGCAGCGAGAGCGCAAGTCCAACTCTCATACTAGCAGCGAGAGCTCCAGTCTCACCGCCTCAAACACCGCTGCCACGGAGGCGGTGTCGGCGGTGACGGAGGTGCCTTCTTTAAGCTCAGCCTCCACTTTGTTGAAGGTCCAGGACTCGAAACTTTTCACTCCTAACCCCAGTTTCGAGCCGGCCGGGTTGCTTGAACAACAAGCCTCGGACTGTGGGATATTCTCGGAGATTGGGAGCTTCACGAGCCTCATAACGGCGTCGAACGACGCATTGCCGTTTGGGTTTAACACTATTTCGGAGATAACGCCGTTTAGGTTGAATCAACAAGGTCATGATCAGTGGCAGCAGCAGGAGCAGAAGGTGATGGGCGGTATGGTTGCTGATCAGGAATTGAAGATGCAGGAGATCACGGGTGGGCTGCTGGATCAGACGGCCCCGGTCGATTTATCGGCGTTGCAGAGCAGATCAAGCGGTGGGGGATTCGGATCGTTTGATTGGCAAGTCAGTGGGGATCAAGGCTTGTTTGATCTTCCTAACACCGTTGATCAAGCATACTGGAGTCACAGTCAATGGGCTGACCAAGACCACCCAAGTCTCTATCTCCCGTAA | MQDMHSIGGSGRLFSGGAGGGGGDRRVRPHHNQHHQHNHQALKCPRCDSLNTKFCYYNNYNLSQPRHFCKSCRRYWTKGGVLRNVPVGGGCRKAKRSKTKPSSDASAPPPQRERKSNSHTSSESSSLTASNTAATEAVSAVTEVPSLSSASTLLKVQDSKLFTPNPSFEPAGLLEQQASDCGIFSEIGSFTSLITASNDALPFGFNTISEITPFRLNQQGHDQWQQQEQKVMGGMVADQELKMQEITGGLLDQTAPVDLSALQSRSSGGGFGSFDWQVSGDQGLFDLPNTVDQAYWSHSQWADQDHPSLYLP* |
| *BpDof 9* | ATGGAAGGGGTAGGGGGTGATCCTAATAACTCAAGGGCTATGTCAGAGAGAAGGGCAAGGCCACAGGAGCACGTAAATTGCCCGCGGTGCAATTCAACCAACACAAAGTTCTGTTACTACAACAACTATAGCCTGACTCAACCAAGATATTTCTGCAAGACTTGCAGAAGGTATTGGACTGAAGGAGGCTCTCTCAGGAACGTTCCTGTTGGAGGAGGTTCAAGAAAGAACAAGAAATTTACATCAGCATCAGCATCAGCAGCAACTTCATCAAAGATTCCTGATCTTAATCCACCAAGCCTCTCACAGTTTTCTTCTCAAAACAATAAGTCCAAGGAAGCAGGACAAGATCTTAATCTGGCTTTCCCAGCTACAGAGAAGTATCATCAAGGCGTTTACCAATATCTTGAAGTGCCCAAAATGGAAGACAACAGCACCAGTACCAATCAACAAAACTCTAGTCATTTTTCAGATTTGATGCTAAGGACTGGAAATATTGCTTCAAGGGGGGGTTTGAATTCTTTTATCCCACCGCCAATGCTTGATTCAAGTACACTCTACTCATCATCCGGGTTTCCTTTCCAAGAATTCAAACCGACCCCTCACCCTGGTTTTTCTGTTGATGGGCTCGGAAGTAGGTATGGGGTTCAAGAGAATAGTACTACTGGGAAGCTTTTGTTTCCTCTTGGAGAATTGAAGCAGATTTCAAGCACAAACGAAGTTGATCAGCAGAGTAAGGGACAGGGGAGTACTTCAACTGGGTATTGGAGTGCAATGTTAAATGGAGGATATTGGTAA | MEGVGGDPNNSRAMSERRARPQEHVNCPRCNSTNTKFCYYNNYSLTQPRYFCKTCRRYWTEGGSLRNVPVGGGSRKNKKFTSASASAATSSKIPDLNPPSLSQFSSQNNKSKEAGQDLNLAFPATEKYHQGVYQYLEVPKMEDNSTSTNQQNSSHFSDLMLRTGNIASRGGLNSFIPPPMLDSSTLYSSSGFPFQEFKPTPHPGFSVDGLGSRYGVQENSTTGKLLFPLGELKQISSTNEVDQQSKGQGSTSTGYWSAMLNGGYW* |
| *BpDof 10* | ATGGACACTGCTCAATGGCCTCAGGAGATTGTGGTGAAACCAATAGAAGAGATAGTCACCAACACTAAGCCTGCAGCAAATTTAGAGAGGAAATTAGCAAGGCCTCAGAAAGAACAAGCTGTAAACTGCCCAAGGTGCAATTCAACAAACACCAAATTCTGTTACTACAACAACTACAGCCTCACCCAGCCAAGATATTTCTGCAAGACATGTAGAAGGTATTGGACTGAAGGTGGATCCCTCAGAAACATTCCTGTTGGTGGAGGCTCAAGGAAGAACAAAAGATCATCATCATCTTCTTCTTCTTCTTCCTCAAAAAAGCTTCCTGATCTTAAGATCCATGAGCGTTCACAAGATCTCAATTTGGCTACTTCTTCCACTACTACTACTACTACAACAAATACATCTCAGATTTCAGCTTTGGAGCTTCTCACAGGGCTTACCTCAAGGAATTTCAATTCTTTTATGCCGATGCCTGTTTCTGCAGATCCAAACACAGTTTACAGTACATCTGGATTTCCCTTGCAGGATTTCAAGCCATCTTTGAATTTCTCTCTGGATGGGCTGGGAAGTGGATATGGGAGTTTTCAGGGGGTTGTTCATGAGAATAGTGGGAGGCTTTTGTTCCCTTTTGAAGATTTGAAACAAGTCTCAAGCACTACAAGCGGTATTGATCATCAGCAAAGCAAAGAGCAAGGAGATTCATCTGGGTATTGGACTGGAATGTTAGGTGGAGGATCATGGTAA | MDTAQWPQEIVVKPIEEIVTNTKPAANLERKLARPQKEQAVNCPRCNSTNTKFCYYNNYSLTQPRYFCKTCRRYWTEGGSLRNIPVGGGSRKNKRSSSSSSSSSSKKLPDLKIHERSQDLNLATSSTTTTTTTNTSQISALELLTGLTSRNFNSFMPMPVSADPNTVYSTSGFPLQDFKPSLNFSLDGLGSGYGSFQGVVHENSGRLLFPFEDLKQVSSTTSGIDHQQSKEQGDSSGYWTGMLGGGSW* |
| *BpDof 11* | ATGTCGTCTGCCCATTCACTTGAAAACATGTTGGTATGCTTAAAAGGGCAGCTTGAGAGGAAACCAAGGCCTCAGCCAGAGCAAGCTCTGAAATGCCCACGATGTGACTCCACAAACACAAAATTCTGCTACTACAACAACTACAGCCTTTCTCAGCCAAGGTACTTCTGCAAATCATGCAGGAGGTACTGGACAAAAGGAGGAACACTGAGGAATGTTCCAGTGGGTGGAGGGTGCAGGAAGAACAAGAGATCATCATCATCAACATCATCAAAGAGGACGCAAGATCAACCACTCACACCCAACAGCAACCCACTAATCAATGGCATCCCATCCATGGCTTATGACTCTCCCAATGATCTCAGCCTTGCATTTGCCAGGCTCCAAAAGCAGTCATGTGGGCAGATGGGATTTGATGATCATCATCACCATGATTTTTCAAGCGGCCATGGTGATATTCTTGGGAATCATGGTATGAACAGTTCAAATCCTGGATTTCTTGATGCTCGAGGTGTAACAGCTCTTAGGAGTGGATTTCATGATGGGCAGAGTAATTTTCAGAATTGGTATTATGGGTATAATGGGAATGGAAACATGGGAGAGGTTGATAATAATGGGGGTGGAGAAATGGTGATGCCATATGATCATCAAGATCAACAAATGAGTAATGCTGCAACGACAACAGCTGTGACAGTGACAACTATGAAGCAAGAATATTGTAGTAATAATGGCAGAGAAAATGATCAGAATAGAGTGTTGTGGGGATTCCCTTGGCAGTTGAATGGGGAAGAAATTGGAAACAATTTGGGTGAGATTGATTCAGGAAGAGAAAGCTGGAATGGAATTGCCCCTTCATGGCATGGCCTTCTTAATAGCCCTCTCATGTAG | MSSAHSLENMLVCLKGQLERKPRPQPEQALKCPRCDSTNTKFCYYNNYSLSQPRYFCKSCRRYWTKGGTLRNVPVGGGCRKNKRSSSSTSSKRTQDQPLTPNSNPLINGIPSMAYDSPNDLSLAFARLQKQSCGQMGFDDHHHHDFSSGHGDILGNHGMNSSNPGFLDARGVTALRSGFHDGQSNFQNWYYGYNGNGNMGEVDNNGGGEMVMPYDHQDQQMSNAATTTAVTVTTMKQEYCSNNGRENDQNRVLWGFPWQLNGEEIGNNLGEIDSGRESWNGIAPSWHGLLNSPLM* |
| *BpDof 12* | ATGCAAGACCCAGCGACATTCCAACCCATGAAACCCAACTTCCCAGAGCCAGAGCAACTCAAATGCCCACGCTGTGGCTCCGCAAACACCAAGTTCTGCTACTACAACAACTACAATCTTTCACAGCCACGCCACTTCTGCAAGAATTGTAGGAGGTACTGGACCAAAGGCGGTTCTCTGAGAAACATCCCAGTTGGCGGTGGAACCCGCAAGAACACAAAGCGGGCATCAAACCCAAAACGCTCCTCTTCATCATCATCATCATCAACGGCGTCAAATTCGGTGGTCCAGAATCCGCCTCCGGAGCACGACCCGACCCGGATGTACGGTTCTGCGGCGGTGGAGCAGGAGCGTCGGATGCTGGATGTCACCGGGAGCTTCAGCTCGCTTCTGGCATCAAATGGGCAGTTTGGAAGCTTCCTGGAGGGTCTGAATCCAAATGGGTCGGGAGTGAAAATGGTGCAAATGGATGAGTTTGCAGAGAATTTAGATTCGGGTCACGGGTTGGACACAGGTTCGGATCGAAACCCGGCAATGGCGGTGCAGAGTAATAGCAACTCGGAGAGCTATTTGGGTTTGGCAAGCGGTGATTCAAGCTGTTGGGCCGGCGGCAATGGGTGGCCCGATCTTGCTATTTACACACCAGGTTCAAGTTTTCAGTAG | MQDPATFQPMKPNFPEPEQLKCPRCGSANTKFCYYNNYNLSQPRHFCKNCRRYWTKGGSLRNIPVGGGTRKNTKRASNPKRSSSSSSSSTASNSVVQNPPPEHDPTRMYGSAAVEQERRMLDVTGSFSSLLASNGQFGSFLEGLNPNGSGVKMVQMDEFAENLDSGHGLDTGSDRNPAMAVQSNSNSESYLGLASGDSSCWAGGNGWPDLAIYTPGSSFQ* |
| *BpDof 13* | ATGGGGTTGAGTTGTAAGCAGGTTTCTAGTGATGGGCTTGATTGGACCCAGAACTTGTTGCAGGAGCTGCCAAAGCCTCCACCAATGAGGCGGCAACAGCAACAAAATCAGCAGCAACAATCCGAGCCGTTGAAGTGTCCACGGTGTGATTCTGCAAACACAAAGTTCTGTTACTACAACAACTACAACAAGTCCCAGCCTCGGCATTTTTGCAGAGCTTGTAAAAGGCACTGGACTAAGGGCGGCACTCTTCGCAACGTTCCTGTTGGCGGCGGCCGCAAGAATAAGCGGACCAAAAAGTCGACCGCCGCCGCATCTGCCAGCACCAGCACCAGCAATACCCTGCAGGCAATTCAAGGTCAGCAGCAGGGGCAGAATCTCCCTGTTTCGCTTGATGATCATCAGAGACACATGTCTGAATTTCTGTACCAGAATTTGCTTCGTCCGCCGTCTTCTCTGCCGCCGCAAAACAACAACAGCAACAACAACATGTGTTTGGGTTCAACTCTGCCTCTTCCTGTAGACCAAAGCCTGCAGTTTCCCTTCTCAAGCTCAAGCTCTTTTAACGCAAACCCATTCTCAGTTTATAATTACTCTGAAGAAATCAATACCAAGGAGGTCCAGGAGCCAACCATTAGCAGCAGTACTGCCACGCAGCCATGGCAAATACCCACAACAAGCAGTGGCATGGACATGCCAACTTACTGGAATTGGGAGGATATTGACCCCTTTGCCTCCACTGATCTCAATCTACCTTGGGATGATTCTGAAATCAAACCATAA | MGLSCKQVSSDGLDWTQNLLQELPKPPPMRRQQQQNQQQQSEPLKCPRCDSANTKFCYYNNYNKSQPRHFCRACKRHWTKGGTLRNVPVGGGRKNKRTKKSTAAASASTSTSNTLQAIQGQQQGQNLPVSLDDHQRHMSEFLYQNLLRPPSSLPPQNNNSNNNMCLGSTLPLPVDQSLQFPFSSSSSFNANPFSVYNYSEEINTKEVQEPTISSSTATQPWQIPTTSSGMDMPTYWNWEDIDPFASTDLNLPWDDSEIKP* |
| *BpDof 14* | ATGCCAACGGAGTCCCCAAACCCAAAGATAAACCTCGCGCGTTTTTATTTCTTCCCCTGTTTGGTTGCCCAGAAAATGCAAGAAAATAAAGACCCCGCCATCAGGCTCTTCGGCAAGAAAATCTCCTTGCCTTCGGACGGAGATACTCTGACCGTCTCCAGAGACGACTTCCCGGTGAGTGCTTCGGAGAAGGAAAAGTGTCGCAGAGTAGGAGGAGCAGAGGAGGAAGGAGGAGGAAGAAGAAACAGAGAAGGTACAGTCCATACAGTGAGTGAGAGTGAGAGGGTTTTACAGTGCGTTGTTTGGAAATTTGAGGACGACCCACTTGTTAGAATGTTAAAAGAGCTGGATCCAACAGCAGCAAAAGGCACTGTGACTACAAAGGAAGATGAAGAGCCTACAGATTTGGAGATATTGCCTGATGCTAATGTGAATCCTAAAACCCCCTCTATAGAGGAAGAGAAAGCAAATGCAAAAACTGCCAAGACGGAGAAAGAACAGAGTGATACAACCAACCCACAAGAGACAACCCTGAAGAAGCCGGATAAAATTCTTCCATGCCCTCGATGCAAAAGCATGGACACGAAATTCTGTTACTATAATAACTACAATGTCCATCAGCCGCGTCATTTCTGTAAATCCTGCCAAAGATACTGGACTGCAGGTGGTACCATGAGGAATGTGCCGGTCGGGGCTGGACGCCGTAAGAACAAGAACTCCGCCTCACATTATCGTCAAATCACCATCTCTGATGCCCTCCAAGTGGCTAGCATTGACGCTCCAAATGGAACTCACTACCCCACATTGAAAAGCAATGGGAGAGTCCTCAGCTTTGGATTAGATGCACCCATATGTGATTCCATGGCTTCTGTTTTAAGCCTTGCAGACAAGAAGGTTTTGAATGGATTTCATAGCTTTGAGGAGCAAAGAATTCCAGTTCCTGGTAAAGGTGGAGAAAATGGTGAAGATTGTTCAAGCGGGTCTGCTATCACAGTTTCAAATTCAATGGAGGGAGGGAAACATTGCCCTCCAGAACCATTTGCGCAATGCATTAATGGCTTCCCTCCTCAAATTCCATGTCTCCCTGGTGTTCCCTGGCCTTATCCATGGAATTCTACAGTAGTACCCCCACCAGCTCTCTGCCCTCCTGGGTTTCCTATGCCATTCTATCCGACAGCTTATTGGAACTGTGGTGTCCCAGGCAGTTGGAACATTCCTTTGCTGTCTCCACAATCTTCTCCGACATTGGGGAAGCACTCAAGAGACGGAGAGATGCTTAAACCAGACAACTTGGAGAAAGAGCAGCATCCAAGACAGGAAAACGGCTGTGTTTTGGTTCCAAAAACTTTGAGGATTGATGACCCAAGTGAAGCTGCAAAGAGTTCTATCTGGGCAACGCTGGGGATCAGGAATGAATCTCTCTGTGGGGGAGGAATGTTTAAGGCCTTTAAATCAAAGAGTGATGAGAAGAGTCACATAGTTGAAACCTCTGCAGTGTTGATGGCAAACCCTGCAGCCTTGTCTAGATCACTCAACTTTCACGAGAGCTCGTGA | MPTESPNPKINLARFYFFPCLVAQKMQENKDPAIRLFGKKISLPSDGDTLTVSRDDFPVSASEKEKCRRVGGAEEEGGGRRNREGTVHTVSESERVLQCVVWKFEDDPLVRMLKELDPTAAKGTVTTKEDEEPTDLEILPDANVNPKTPSIEEEKANAKTAKTEKEQSDTTNPQETTLKKPDKILPCPRCKSMDTKFCYYNNYNVHQPRHFCKSCQRYWTAGGTMRNVPVGAGRRKNKNSASHYRQITISDALQVASIDAPNGTHYPTLKSNGRVLSFGLDAPICDSMASVLSLADKKVLNGFHSFEEQRIPVPGKGGENGEDCSSGSAITVSNSMEGGKHCPPEPFAQCINGFPPQIPCLPGVPWPYPWNSTVVPPPALCPPGFPMPFYPTAYWNCGVPGSWNIPLLSPQSSPTLGKHSRDGEMLKPDNLEKEQHPRQENGCVLVPKTLRIDDPSEAAKSSIWATLGIRNESLCGGGMFKAFKSKSDEKSHIVETSAVLMANPAALSRSLNFHESS* |
| *BpDof 15* | ATGATGTCACCGGATAATAACATTCCGGCAAAGTCGGTTGTCAGAGACGACAGCCAAGGCTCCGGCAGCCGCAAAACTGGGTCAGCGAGGCCACCAGAGCAAGGCCTAAAGTGCCCACGATGCGACTCACCCAACACCAAATTCTGCTACTACAACAACTACAGCCTCACGCAGCCAAGGCATTTCTGCAAGACTTGTAGAAGGTACTGGACAAAAGGCGGGGCTTTGCGCAACGTTCCCATAGGCGGCGGTTGCCGGAAAAACAAAAAGGTTAAGTCATCCTCGAGGCTCTCCGGCGCCGACTCCAAGGACTCAGCCTCATCTTCGGAGATCGGCGGATTGAAGTTCTTCCACGGTCTATCCCCCGCCATGGATTTTCAGCTTGGTGGGTCGTTATCATCATTCCCTAGACTCCACCCTCCAACAACTGGTATTAATTACAACCAGTTTTCTTCCTTTGGAGACGTTTCGGGTACTACTTTTGCTGCTGGTTCCGGAATTACCCTCGATCCATCTGGAACCTCTAATTCTCTGATGGGTTTTAACTACCCGCTTTCTTCGGCAACTGCCGGTGGTAGTTACAGCCCTGCAGTTCAGAACACGAGCTCCATGAACGTTCACACCAATCTCGCTTCTTCTATCGAGTCTCTGAGTACTATAAACCAAGACTTGCACTGGAAGCTTCAGCAGCAGCGGCTTGCGGTGCTATTTGGCGGAGAGACTCACAAAGATCATCAGAAACCGCAACCCATTGTTTTCCAGAACCTGGAGATTTCGAAACCAGAGGCTTGCGGCGTTAATGGTAATAGTTCAAGGAAAGAAGGTACAGCTGGCGATCAGATAGCGACGGAGTGGTTCTTCGGGAACTCTTACGGGMPSDSSEHRRPPKTTHNPGAPPPEQEQLPCPRCDSTNTKFCYYNNYNFSQPRHFCKSCRR  YWTHGGTLRDIPVGGGSRKNAKRSRTANVTNIVSSAVTSHVDPLSATPVLLPLSASQGSS  VQFGVGGNGGDVKGNAGVCGSFTSLLNTQGPGFLALGGFGLGLGPGFEDMGFGLGRGIWA  FPGVGDGGAGGGGNGGATGMGNTWQFENGESGFAGGDCFSWPDLAISTPGNGLK*CCGGTGACCCCTACTCCGACCAACAGTGGCGGCAACGGTGGACACGATAACGCGAGCGGTTGGAATAATATTGGAATTCAAACATGGGGTGACTTGCAACAATATAGCACTTTGCCCTAG | MMSPDNNIPAKSVVRDDSQGSGSRKTGSARPPEQGLKCPRCDSPNTKFCYYNNYSLTQPRHFCKTCRRYWTKGGALRNVPIGGGCRKNKKVKSSSRLSGADSKDSASSSEIGGLKFFHGLSPAMDFQLGGSLSSFPRLHPPTTGINYNQFSSFGDVSGTTFAAGSGITLDPSGTSNSLMGFNYPLSSATAGGSYSPAVQNTSSMNVHTNLASSIESLSTINQDLHWKLQQQRLAVLFGGETHKDHQKPQPIVFQNLEISKPEACGVNGNSSRKEGTAGDQIATEWFFGNSYGPVTPTPTNSGGNGGHDNASGWNNIGIQTWGDLQQYSTLP* |
| *BpDof 16* | ATGCCTTCGGACTCCAGCGAGCACCGCAGGCCACCCAAGACCACCCATAACCCGGGAGCCCCACCACCAGAACAAGAGCAACTGCCCTGCCCGCGCTGCGACTCCACCAACACCAAATTCTGCTACTACAATAACTACAATTTCTCGCAGCCCCGTCACTTCTGCAAGTCCTGCCGTCGCTACTGGACTCACGGCGGGACTCTCCGCGACATCCCCGTCGGCGGTGGCTCCCGCAAGAACGCCAAGCGCTCCCGCACAGCCAATGTCACCAATATCGTCTCCTCCGCCGTCACTTCTCACGTCGACCCTCTATCCGCCACCCCGGTCCTGCTCCCTCTTTCGGCCAGCCAGGGATCGTCCGTACAGTTTGGGGTTGGTGGTAATGGGGGTGATGTGAAGGGCAATGCGGGTGTTTGTGGGAGTTTCACGTCTCTGCTGAACACTCAGGGGCCGGGCTTTTTGGCGCTCGGTGGGTTTGGGCTTGGGCTTGGCCCTGGGTTTGAGGATATGGGGTTTGGGCTTGGGAGAGGGATCTGGGCTTTTCCAGGGGTGGGAGACGGTGGTGCCGGTGGTGGTGGCAATGGCGGTGCAACGGGAATGGGGAACACGTGGCAGTTCGAAAATGGAGAGTCTGGGTTTGCTGGTGGAGACTGCTTTTCTTGGCCGGATTTGGCAATTTCAACCCCTGGAAATGGTCTCAAATGA | MPSDSSEHRRPPKTTHNPGAPPPEQEQLPCPRCDSTNTKFCYYNNYNFSQPRHFCKSCRRYWTHGGTLRDIPVGGGSRKNAKRSRTANVTNIVSSAVTSHVDPLSATPVLLPLSASQGSSVQFGVGGNGGDVKGNAGVCGSFTSLLNTQGPGFLALGGFGLGLGPGFEDMGFGLGRGIWAFPGVGDGGAGGGGNGGATGMGNTWQFENGESGFAGGDCFSWPDLAISTPGNGLK* |
| *BpDof 17* | ATGGCTAACGTTAACGGCGAAGATGTGCCCGGAAATGGAATCAAGCTATTTGGGGCGACGATAACATTGCAGAATAGACAGGTGAAGGAAGAACAGAAAGGGGCTGGTGATGATGATCAGACGGTGGAGAAGAGGCCAGATAAGATCATACCGTGCCCAAGATGTAAGAGCATGGAGACTAAGTTTTGTTACTTCAACAATTACAACGTCAACCAGCCTAGGCATTTCTGTAAGGGCTGCCAGAGGTACTGGACGGCCGGTGGGGCTCTCCGGAACGTGCCGATCGGCGCCGGACGCCGGAAAACCAAGCCACCGCCTCGTGGGTTCTCTGAGGGTTGCTTGTATGATGCTGATGGGCTCCACCAGTTTGACTTTGATGGAGTGGTCGAGGAGTGGCATGTCATGGCCGCCCAGGGCGGTTACCGGCATGTTTTTCCGGCGAAGCGGCAGAGGAGTAGCTCAGGTGGTCAAGCATGCCCTTGA | MANVNGEDVPGNGIKLFGATITLQNRQVKEEQKGAGDDDQTVEKRPDKIIPCPRCKSMETKFCYFNNYNVNQPRHFCKGCQRYWTAGGALRNVPIGAGRRKTKPPPRGFSEGCLYDADGLHQFDFDGVVEEWHVMAAQGGYRHVFPAKRQRSSSGGQACP* |
| *BpDof 18* | ATGCAGCAGGAGAGAGATGGAGAAGGAGGAGAAGACGTGAAGCAACAAAATCATCAAGATCAGCGGAGAGTGAAGGCGATGCAAGGTGAAAATCAGCAGCAAAATCAGCAGCCACAAAAATGCCCTCGCTGTGAGTCTCTCAACACCAAGTTTTGCTATTACAACAACTATAGCCTGTCTCAGCCGCGTTACTTCTGCAAGACTTGTAGAAGGTACTGGACTCAAGGAGGTACCCTCAGAAACGTCCCCGTCGGCGGCGGTTGTCGGAAGGGAAAGCGTGCCAAAACAACCTCCTCATCTTCCGGTGAGAATTCACGGTCGTCGACTCAGCAGCCACAACAACAAAATTTGACAAACCCACCACCTACAAATACAATCCCCTCTAACCCAGTTATCCAAACCAGCCCCTCGGCTCTAAGAACGACTAAAGAATTAGGATCGATCCCGTCAATTGCGCCTTACTATGCGGGAGGTGGTTATTTGTCTTCCTTGGGGGCAATTCAATCTTTCAGTCAATCACTCCATCATGTTGGGGGTTCTTCAAATATGGATCTGCTTCAGGGCTTTACTCTCCCTTCTTTTGGGTCACAACAGCAGCAATTTTATCAAATGGGTAACAGAGATAGAAGCATGGAACATCTCTATCCGAGGACTGAAGAAAATTTGATTCAATCCAGCAGGCCTACCAGTAGTTCCCAGCAGGATGATTGGCATCAGGGCTTTATGATGAGCAATACTAATCCCCCGCCGTCTGATCCCGCTTTGTGGAGTTTTAGCACCATTACCAGTACAACTGGAAACTCCAACAGCAATAACAACAACGCTGGCTCTACCTTTAATATTCCAAGTCAGTGGCCTAATCTCCCAGGATATGGCCCTCCTCCGTGA | MQQERDGEGGEDVKQQNHQDQRRVKAMQGENQQQNQQPQKCPRCESLNTKFCYYNNYSLS  QPRYFCKTCRRYWTQGGTLRNVPVGGGCRKGKRAKTTSSSSGENSRSSTQQPQQQNLTNPPPTNTIPSNPVIQTSPSALRTTKELGSIPSIAPYYAGGGYLSSLGAIQSFSQSLHHVGGSSNMDLLQGFTLPSFGSQQQQFYQMGNRDRSMEHLYPRTEENLIQSSRPTSSSQQDDWHQGFMMSNTNPPPSDPALWSFSTITSTTGNSNSNNNNAGSTFNIPSQWPNLPGYGPPP* |
| *BpDof 19* | ATGTTGGGCAACTGTGAGAAGATGGTTCTCATCCCTCCCACTACAAACGAATGGTCACAGCAGAACCAGATAGATGATCCAAAGGCCTTGATGGCTTCGACTGGCAGGGGAATGGAAAAACCAGACCAACAACAACAGCAGCAGCAACAACAACAACAACAACAACAAGCGCTGAGATGTCCACGCTGTGATTCATCAAACACAAAGTTCTGCTACTACAACAACTATAGCTTGTCACAGCCAAGGCATTTCTGCAAGGCCTGCAAGCGTTACTGGACAAGAGGGGGAACTCTAAGAAACGTTCCTGTAGGCGGTGGGTGCAGGAAAAACAAGCGCGTGAAGAGGCCTGCAACGGCTAATTCCGCCATTGATGGATCCGGAGCTTCTTCTGCAGCTCCACCAGACCCTCCATCGCAACCCCACATGGATACATCAAATCATATGAATCCTTTGTTTTATGGTTCTGATCATGTGAGATCATCAGTTCCATTTCCCACCAGGTTCAATATTAATCCCAGAGTTTCAAGTGAAGCTGTTTCTGGGTATGATCTCCAACCCCAGATGAATGCTCTTGGATTGGGGTTTTCTTCTGCGATTATGTCTAATGATATTGGTAGTGAGAACGATTACCGAAATGGGTTTAATCTCTCAAGTACTAATTATTCCATTTTCGGCGGCGGCTCAGCTAATTCTCCAACCATGGCTTCCCTGCTAAGCTCTAGCTTTCAGCAACAGAAATTCGTCAATGGAGGTATCAAAGACGGTACTACTCGAGCAGTACCGAATCATTTTCAAGGCTTTGGACCGTTTGAAGGGTTGCAAATAATGGGTCATGGTACGGAAGCTGGGATTTCCATGAAAGAAGTGAAAGTAGAAGATCATCATCACAGCCAAAACAGGTCGGATTGGAATCAAATGGAGCAAGTCGGCTTGTCGGATCCTACTCTTTACTGGAATGCAACAAGTAATATTGGCGTTTGGAATGATCCTGGAAACAATATCGGGTCTTCGGTCACTTCTCTGATTTAA | MLGNCEKMVLIPPTTNEWSQQNQIDDPKALMASTGRGMEKPDQQQQQQQQQQQQQQALRCPRCDSSNTKFCYYNNYSLSQPRHFCKACKRYWTRGGTLRNVPVGGGCRKNKRVKRPATANSAIDGSGASSAAPPDPPSQPHMDTSNHMNPLFYGSDHVRSSVPFPTRFNINPRVSSEAVSGYDLQPQMNALGLGFSSAIMSNDIGSENDYRNGFNLSSTNYSIFGGGSANSPTMASLLSSSFQQQKFVNGGIKDGTTRAVPNHFQGFGPFEGLQIMGHGTEAGISMKEVKVEDHHHSQNRSDWNQMEQVGLSDPTLYWNATSNIGVWNDPGNNIGSSVTSLI* |
| *BpDof 20* | ATGCCCAAGGCTTTGATAGAGAGGCGGCTGAGGCCACCACATGACCAGGCGCTCAAGTGCCCACGGTGTGAGTCAACACACACAAAATTTTGCTACAACAACAACTACAACCTCTCTCAGCCAAGGTACTTTGCAAGACCTGTAGAAGGTATTGGACCAAAGGAGGCACCCTTAGGAACATTCCTGTCGGCGGTGGATGCTAGAAAGAATAAGAAGTTTTCTGTTAAAAAATCCAACGATCAACCCATGACCCACCAAAACCATCCCGGGTCATCATCGTCTCACAACCCAACTGATCTTCAGCTTTCTTTTCCGGAGGCCCAACTTTCCCACCTTACCAATATACTTGGCACACACGGTACACTTGGAAACCCTAATTATATGGACGCCAGGTATCATGGTATGATGGAGAACCCTAGGCCTATTGATTTTATGGAGAGTAAGTTGGATAGAAATTATGATTTTATGGGGAATGGTGATCATCACATGGGATCGTGGACCGGCATGATGAACGGTTACGGACCGTTGACAACAAATCCATTAGTCTAA | MPKALIERRLRPPHDQALKCPRCESTHTKFCYNNNYNLSQPRYFARPVEGIGPKEAPLGTFLSAVDARKNKKFSVKKSNDQPMTHQNHPGSSSSHNPTDLQLSFPEAQLSHLTNILGTHGTLGNPNYMDARYHGMMENPRPIDFMESKLDRNYDFMGNGDHHMGSWTGMMNGYGPLTTNP  LV* |
| *BpDof 21* | ATGTCTCAGTTAAAAGACCCGGCGATAAAGCTGTTCGGTAAGACGATTCCTTTGCCACTGAAGCAGAGAAGTTGTGCTAATGAGCTATGTGGAGCTGAAGATCGCTTTGACCAAAATCTTCTTTCTTCAACCACTTCTTCCCTCGAAGAGAATTGTGGCAGAGAAGGTGCTGTGCAAGAAGGGGATCAGGTACACTTGGGAAGAGAACCCACCGAAGATAAACAGGAAGATTGTAACTCATATCAGACCGTAGAAGACTCTAAAGATCATTCAACATCATCAGGAATTAGCGAGAACCCCCAAACTCCCTTGATTGACAGAGAAACTTCGTCACTAAAAGCCTCCAAGAATGGAGAAGAGAGCGAGACAACTATCTCACAAGAAAAGACGCTGAAGAAGCCTGACAAAATACTTCCATGTCCCCGGTGTAATAGCATGGATACCAAGTTCTGTTACTACAACAATTACAATGTCAACCAGCCCCGTCATTTCTGTAAGAACTGTCAAAGATACTGGACTGCTGGTGGAACTATGAGGAATGTACCCGTAGGTGCTGGTCGCCGTAAGAACAAGAACAACTCTAGTTCACACTATCGTCACATTATAGTTTCAGAGGCTCTTCAAACGGCCCGAGCTAATGGAGTACACAGCTCTGCAATGGGAAAAAATGGAACCGTCCTCACCTTTGGCTCGGATTCTCCTCTTTGTGAATCCATGGCTTCTGTATTGAACCTTGCAGAGAAATCACAAAACCATGTTCAAAATGGGTTTCATAGACCTGAGCAAAGAACTCTTGTTTCTAGAGGAGGAGATAATGGGGATGATCACTCAAGTGGATCCTCTATCACAGCTTCAAATTCAACTGAGAGGGAAGGTTTGCAAGAGTCGGCAGTTAAAAATTATCAAGGGTTTCCTCCTCAAGTACCGTGCTTTCCAGGGACCCCTTGGCCTTTACAGTGGAACTCACCTCACTGGGGCTCTGTAATGCCTCCATCTGCTTTGTGCCCATCAGGATATCCACTACCGTTCTACCCTGCTCCAGCATTTTGGGGTTGTACTGTACCAGGCTCTTGGAATATACAATCTCTTTCCCCACCATCCTCTTTTCTTAGCCACAGTGGCCAAAGCTCTGCTCCTAATTCCCCAACCTTGGGCAAACATTCAAGGGATGGGAGCATCCTTAATCCAGCCAACTCACAGCAAGAAGAGTCTGTTAAAGAGCAAAGTTCAGAAACATGTGTTTGGATTCCTAAAACTTTGAGAATTGACGATTCCAGTGAAGCCGCAAGGAGCTCTATATTGGCAACACTTGGGATCAGGAATGAGAAGACTAATCCTGCCAATGGTGGAAGTCTCTTTAAGGCATTCCAATCAAAGGGTAATGACAAAAGTCATGTGGTCGAGACATCTCCAGTGCTGCAAGCCAACCCTGCAGCCTTATCCCGGTCACTAAATTTCCATGAGAGCACATAA | MSQLKDPAIKLFGKTIPLPLKQRSCANELCGAEDRFDQNLLSSTTSSLEENCGREGAVQEGDQVHLGREPTEDKQEDCNSYQTVEDSKDHSTSSGISENPQTPLIDRETSSLKASKNGEESETTISQEKTLKKPDKILPCPRCNSMDTKFCYYNNYNVNQPRHFCKNCQRYWTAGGTMRNVPVGAGRRKNKNNSSSHYRHIIVSEALQTARANGVHSSAMGKNGTVLTFGSDSPLCESMASVLNLAEKSQNHVQNGFHRPEQRTLVSRGGDNGDDHSSGSSITASNSTEREGLQESAVKNYQGFPPQVPCFPGTPWPLQWNSPHWGSVMPPSALCPSGYPLPFYPAPAFWGCTVPGSWNIQSLSPPSSFLSHSGQSSAPNSPTLGKHSRDGSILNPANSQQEESVKEQSSETCVWIPKTLRIDDSSEAARSSILATLGIRNEKTNPANGGSLFKAFQSKGNDKSHVVETSPVLQANPAALSRSLNFHEST* |
| *BpDof 22* | ATGGCTTTCTCATCTGTTCCAGTCTATTTAGATACTCACAATTGGCAGCAGCAACCAAATCATCAACAAGGAAGTGATGACAATCAAAACCCACTTCTCACACCACCACCCCATCATGCCGGCGGCGGCGGCGGTGCCGGCTCGATCAGGCCCGGTTCAATGGCTGATCGAGCCAGGTTAGCCAAGATACCCCAGCCGGAAACAGCTCTCAAGTGTCCCAGGTGTGATTCCTCCAATACTAAGTTTTGCTACTTCAATAATTACAGCCTTACTCAGCCCCGACACTTCTGCAAGACATGTCGGCGCTACTGGACTAGAGGAGGTGCCCTTCGGAATGTTCCGGTGGGGGGCGGTTGCCGTAAAAACAAGAAGAACAAAAGCAACCGCAATAAATCTCCAGCTGCAGCTGCGGCGATTCCCTCTAGCAGTGCAATTCCCTCTGCCTGTACTTCAGAGATAAATATGGGTCATCATAATTTGCCTCAGCAACCTCCTCAATTACCCTTCATGGCCTCTTTACAGAATCTTAGTCGGTATGGTGTGGGAAATAATATTGGCTTAAACTTCACTGACATTGATGTCAGAAGCTTGCCTTCATCTGATGTCAGAAACTCGAGATATGACCTTTTTATGAAGTCTGCTGGTCTGATTGAAAACCACCCTGCATCACAGACTGATCATCTGGGGTATCAGATTGGAAGCGGTTCAGGAGGAGGAGGAGGAGTGGATCATCAGTGGCGGTTGCAGCAATTCCCTTTCATGGGTGGATTTGAGTCATCCCCAGCTTTATACACAATTCAAAGTGAAGGAGGAGTCCAAGTACCATCTCCTTTAGTTGGAGATAGTAGTGAGCTTAGGGTTTCTCAGCTTCCTCCAGGGATGAAAATGGAAGAAAGAAATTTATCAGGTCTCAACTGGGGAGGAAATGCATGGACAGATTCATCCACCCATCTGTTGTAA | MAFSSVPVYLDTHNWQQQPNHQQGSDDNQNPLLTPPPHHAGGGGGAGSIRPGSMADRARLAKIPQPETALKCPRCDSSNTKFCYFNNYSLTQPRHFCKTCRRYWTRGGALRNVPVGGGCRKNKKNKSNRNKSPAAAAAIPSSSAIPSACTSEINMGHHNLPQQPPQLPFMASLQNLSRYGVGNNIGLNFTDIDVRSLPSSDVRNSRYDLFMKSAGLIENHPASQTDHLGYQIGSGSGGGGGVDHQWRLQQFPFMGGFESSPALYTIQSEGGVQVPSPLVGDSSELRVSQLPPGMKMEERNLSGLNWGGNAWTDSSTHLL* |
| *BpDof 23* | ATGGAGGGTGGTAATAATTCAAGGGCTATGAGGGCAAGACCACAAAAAGATCAGGCTTTGAACTGTCCAAGGTGCAATTCAACAAATACCAAATTTTGTTACTACAATAATTACAGTCTCTCTCAGCCAAGATATTTCTGCAAGACTTGTAGAAGGTATTGGACTGAAGGTGGGTCTCTAAGAAATGTTCCTGTGGGAGGAGGCTCAAGGAAGAACAAGAGATCATCACCTGCAAAGAAGCTTCCAGATGACCATCTTACCCCTCACTCTGCTTCTCAAAACCCTAAGATCCCTCAAGGGCAAGATCTAAACCTAGCTTACCCACCAAGTTTCCAAAACCCTAGCCCCTCGTCCACGTCATCTCAGCTCTCAGCCATGGAACTTCTCAAGAGTAGTACGATGCCAGGTGGGTCAAGAGGGTTGAGTTCTTTCATGCCGATGCCAGCTGTAATATCCGATTCGTATTCGTCCGGGTTTGGTATGCAAGAATTCAAACCGAGTACGCTCAATTTTTCGCTCGAGGGTTTTGAAGGCGGGTATGGGAGTCATCTTCAGGGAGTGCAAGAGAGTGGCGGTGCAAGGCTGTTGTTTCCGATTGATGATTTGAAACAGGTTCCGCCGACGAGCGAATTCGGGCAGAATAGAGGGCAGGGAGCAGATTCTGCAGGATATTGGAATGGGATGTTAGGAGGAGGATCATGCAAAGAGTTCAGTGATGATGATGATCATGTTCTTGATGATGATATTTCTTGCTACAGATTAATAATGTTCCATGATGTTTTAGGTCAGAAGTGTCAGGTCTTTTATCAGCACTATCTGGGTCGTAGAGATTCTTCCACCCATTCAAGCACCTTCTTGAAGCAATGGGATGTGAAGAACTACAAGAATGACTAG | MEGGNNSRAMRARPQKDQALNCPRCNSTNTKFCYYNNYSLSQPRYFCKTCRRYWTEGGSLRNVPVGGGSRKNKRSSPAKKLPDDHLTPHSASQNPKIPQGQDLNLAYPPSFQNPSPSSTSSQLSAMELLKSSTMPGGSRGLSSFMPMPAVISDSYSSGFGMQEFKPSTLNFSLEGFEGGYGSHLQGVQESGGARLLFPIDDLKQVPPTSEFGQNRGQGADSAGYWNGMLGGGSCKEFSDDDDHVLDDDISCYRLIMFHDVLGQKCQVFYQHYLGRRDSSTHSSTFLKQWDVKNYKND* |
| *BpDof 24* | ATGGATACAGCTCAGTGGCCACAGGGAATTGGAGTAGTCAAACCCATGGAGGGTGGTAATAATTCAAGGGCTATGAGGGCAAGACCACAAAAAGATCAAGCTTTGAACTGTCCAAGGTGCAATTCAACAAATACCAAATTTTGTTACTACAATAATTACAGTCTCTCTCAGCCAAGATATTTCTGCAAGACTTGTAGAAGGTATTGGACTGAAGGTGGGTCTCTAAGAAATGTTCCTGTGGGAGGAGGCTCAAGGAAGAACAAGAGATCATCACCTGCAAAGAAGCTTCCAGATGACCATCTTACCCCTCACTCTGCTTCTCAAAACCCTAAGATCCCTCAAGGGCAAGATCTAAACCTAGCTTACCCACCAAGTTTCCAAAACCCTAGCCCCTCGTCCACGTCATCTCAGCTCTCAGCCATGGAACTTCTCAAGAGTAGTACGATGCCAGGTGGGTCAAGAGGGTTGAGTTCTTTCATGCCGATGCCAGCTGTAATATCCGATTCGTATTCGTCCGGGTTTGGTATGCAAGAATTCAAACCGAGTACGCTCAATTTTTCGCTCGAGGGTTTTGAAGGCGGGTATGGGAGTCATCTTCAGGGAGTGCAAGAGAGTGGCGGTGCAAGGCTGTTGTTTCCGATTGATGATTTGAAACAGGTTCCGCCGACGAGCGAATTCGGGCAGAATAGAGGGCAGGGAGCAGATTCTGCAGGATATTGGAATGGGATGTTAGGAGGAGGATCATGGTAA | MDTAQWPQGIGVVKPMEGGNNSRAMRARPQKDQALNCPRCNSTNTKFCYYNNYSLSQPRYFCKTCRRYWTEGGSLRNVPVGGGSRKNKRSSPAKKLPDDHLTPHSASQNPKIPQGQDLNLAYPPSFQNPSPSSTSSQLSAMELLKSSTMPGGSRGLSSFMPMPAVISDSYSSGFGMQEFKPSTLNFSLEGFEGGYGSHLQGVQESGGARLLFPIDDLKQVPPTSEFGQNRGQGADSAGYWNGMLGGGSW* |
| *BpDof 25* | ATGCTGGCGACCACGACGGCAGCAACACCAGAGAGTTCCGATGATGACGCGCCAACCTATCACCGAAACCACTCACCAAACGTTCACCGAGAGAGGCGCACCACCACCAACAGAGAACCGGCCACCACAGACGGCACCAACCACCACAGAACTCGCCGACGAAACCAACCGACCACCCTGAAACATCTATCTGAAATCAGGGGTTCCACCTACGACACCAACCGCACCACTGACGGAGCAAAACATGGCGATGACGACGATATCTCACCAACGACGGTGATCTCGTCGCCGCCGTCGCCGGACACGGGCATGAAGCCCAGTGCCTTGGTCTCCTTCGAGATGCAGCAAAATCAAGGCTTCGGAGGGATTTTGTGTTTTATGTATGGATCTGAAGAAGCAACAAAATCTGGAGTCAATGACCTTCTCGAGGAAAAGTCTTGTGAGCAGGATATCCCTTCCAGATTAGATAATCCAAAAGATATCGGCTGCCAAAATCCTCCACAGGAGAAGGTTTTAAAGAAGCCAAACAAGGTCCTCCCTTGTCCTCGCTGCAAAAGCTTGCAGACAAAGTTTTGCTATTTCAATAATTGCAATGTAAACCAACCAAGATACTTTTGCAAGAATTGCCAGAGATATTGGACAGCTGGGGGAACAGTTAGAAATGTACCTGTTGGGGCTGGAAGGCGCAAGAATAAGCATTCATCTTCACAGAGCCATCAGGTAATGGACGAAGTCCCAAAGTTTAGCAAAGATGCTCCTCTATGTGAATCCATGGAAACTGTGCTTAATCTCATAGACCCAAATAAAAGTGTTGAAATTTGGTCTTCGGCTGCTGAGGGTGATGGTGAAGAGCCCTTATCATCTGTTTCTTCCTCAGCAGCTGCTACTTCCCAGGAAAGTGAATTTCCAGAGAAGGCAATCAAGCAGGTTGCATCTTCAGGACATTGCAACAGTTTCAATCCAATGCATACACTGCAGTCTTACCCTGCTTCCCAATGTGTTTACCATTGGAACCCAGGTTGGAATGCAATGTTGTTTGGGCCCAGTAGCAGTATCTCTAATACTGCTCATATGGTTTCTCCACCTATGGTGCCTGTTACTGGCTTTTGTGAGCCAAGCATCACATTCCCATTTCTTCCTGCCTCATACTGGGGTTGCGTGCCATTGGTTGGATCTACTTGCAGCTCATCAGCTTCATCTTCTACGAGCAACAGCAGCTGTTCAGGTAATAGCTCGCTGGCCTTGGGGAAGCATTCAAGGGATGCAAATTCACAGGCGGAAGAAACAAAGGAACAAAGCCTTTGGGTGCCCAAGACACTGAGAATTGATGACCCAGAGGAGGCTGCAAAGAGTTCCATATGGTCTACTTTGGGTATTAGACTTGGCAAGGACAAACCCAACATCAGAGATGGTATTTTTAAAGCTTTCCAATCCAAATCAGATAGTAGTTCCCCTACATCAGATGCTGATCAAATTTTAAAAGCAAACCCAGCAGCTTTTTCTCGCTGCCAATCGTTCCAGGAAAGCACTTAA | MLATTTAATPESSDDDAPTYHRNHSPNVHRERRTTTNREPATTDGTNHHRTRRRNQPTTLKHLSEIRGSTYDTNRTTDGAKHGDDDDISPTTVISSPPSPDTGMKPSALVSFEMQQNQGFGGILCFMYGSEEATKSGVNDLLEEKSCEQDIPSRLDNPKDIGCQNPPQEKVLKKPNKVLPCPRCKSLQTKFCYFNNCNVNQPRYFCKNCQRYWTAGGTVRNVPVGAGRRKNKHSSSQSHQVMDEVPKFSKDAPLCESMETVLNLIDPNKSVEIWSSAAEGDGEEPLSSVSSSAAATSQESEFPEKAIKQVASSGHCNSFNPMHTLQSYPASQCVYHWNPGWNAMLFGPSSSISNTAHMVSPPMVPVTGFCEPSITFPFLPASYWGCVPLVGSTCSSSASSSTSNSSCSGNSSLALGKHSRDANSQAEETKEQSLWVPKTLRIDDPEEAAKSSIWSTLGIRLGKDKPNIRDGIFKAFQSKSDSSSPTSDADQILKANPAAFSRCQSFQEST* |
| *BpDof 26* | ATGAGTTGTGGAGGAGATATCTCTGAGGAGGCGACTAAGGATCCTGGCATAAAGCTCTTTGGCAGAAAGATCCCTATGCCGGAATGTCGGATTCCGGCCAGGTCGGACGTCATGAAGCAGCTGAATGAATTTAATGCCAGTGTCAGTAAATTGACTGCTATCTTAGTCAATATGGATGAAGAAAAGAATTACCATTTTGCTTTGTTCCATGCAACACTCGTTGGATGGTTAGTGGAGAGCTTTTATGCAGAAAACTTAGGGGAGCCACATAAGTTCTCTGCTTTAGGAAATAGGAATGAAGAGCCCCAAACTCCTGTGCAGGTTGAGGCACCAGTATATCCTACGCCCAAGCAAGGGCAGGTAGAGACCAATACTACAGAGCAAGAGAAAGTGTTTAAAAAACCAGACAAGGTACTTCAATGTCCAAGATGCAACAGTTTAGACACAAAATTTTGCTATTTCAATAACTACAATGTCAGCCAACCTAGGCATTTCTGTAAGAATTGCCAGAGATATTGGACGGCTGGTGGAACCATGAGAAATGTTCCAGTGGGTGCTGGGAGACGAAAGAACAAGCACTTAGCCTCTCAATACCATCAGATAATAGTATCATCTGATGGAGTAGCAATGACAAGATCGGAAATGACAGACTCAGCCAACCACCAGCTTCTGTCGTGTGGTGAATCTTCAACGGTAAATGGGACACTCCTAAAATTTGGTCCTGAAGCACCTCTTTGTAGATCGATGGAGACTGTTCTTAATTTAAGAGACCAGAGAAGATGTGTTGAGATGGGTTCTTTAAATGTCGAAGAAAATGAAGAGCCTTCCTCATGCATATCATCCATGACAGCTTCCAGTAATCAGGGAAATCAATTACCTGAAAATACTGAGCAAAGGGAGCGAGTCTGTTTGCCAGGATCTCGTAATGAGCTTAATACACCACATCCTCTGCATTGTTACCCTGTTCCTCCATGGAGTTTCCCTTGGAGTGCAGGTTTGCACAATGACGCCTCTGTTGCAAAAGCAGAAAATGCTTCTGAGTGCATTTCCGTACCAAATGGCAGCGATCCAAATCCTGTTCAGTGGTACCCTACACCAATGCTTGCAGTTCCAGCCGGCTTTTGTCCTCCGAGCATTCCTTTACAATTTGTACCAGCATCATATTGGGGTTGTATGCCTGTTTGGACTCCCGGAGCTGGATCTAGTGGTTTATCTCCATCATCCTCTGCGAGTAACAGTTGCTGCTCTGGCAATGGCTCACCAACCCTCGGCAAGCATTCCAGAGATGCAAAATTTACAGATGAAGAGAGATCAGAAACATGTATTTTTGTTCCAAAGACACTGAGAATTGATCACCCAGATGAGGCTTCAAAGAGTCCTATATGGGCCACATTAGGAATTAAGCCTGACCAGAAGCAATCTATATCGAAAGGTACTATTTTCAGAACATTTAAATCAAAGGCAGAAGAGATGGAGCATTTGGCTGAAGCGTCTGAGATCTTGGGTGCAAACCCTGCAGCTCTTTCTCGCTTTCATACATTTCAAGAGAGCAACTGA | EKNYHFALFHATLVGWLVESFYAENLGEPHKFSALGNRNEEPQTPVQVEAPVYPTPKQGQVETNTTEQEKVFKKPDKVLQCPRCNSLDTKFCYFNNYNVSQPRHFCKNCQRYWTAGGTMRNVPVGAGRRKNKHLASQYHQIIVSSDGVAMTRSEMTDSANHQLLSCGESSTVNGTLLKFGPEAPLCRSMETVLNLRDQRRCVEMGSLNVEENEEPSSCISSMTASSNQGNQLPENTEQRERVCLPGSRNELNTPHPLHCYPVPPWSFPWSAGLHNDASVAKAENASECISVPNGSDPNPVQWYPTPMLAVPAGFCPPSIPLQFVPASYWGCMPVWTPGAGSSGLSPSSSASNSCCSGNGSPTLGKHSRDAKFTDEERSETCIFVPKTLRIDHPDEASKSPIWATLGIKPDQKQSISKGTIFRTFKSKAEEMEHLAEASEILGANPAALSRFHTFQESN* |
