# Supplementary material for: Genome-wide analysis of BpDof genes and the tolerance to drought stress in birch (Betula platyphylla)
Source: PeerJ. 2021 Aug 24;9:e11938. doi: 10.7717/peerj.11938 (PMC8395574; doi:10.7717/peerj.11938)
Supplement: Supplemental Information 2 [file peerj-09-11938-s002.doc]

**Table S2.** Gene codes and amino acid sequences of AtDof TFs.

| Gene code | Other names | amino acid sequences |
| --- | --- | --- |
| AT1G07640 | OBP2 | MAFPSNWSQPTNSNHQHHLQHQLNENGSIISGHGLVLSHQLPPLQANPNPNHHHVATSAGLPSRMGGSMAERARQANIPPLAGPLKCPRCDSSNTKFCYYNNYNLTQPRHFCKGCRRYWTQGGALRNVPVGGGCRRNNKKGKNGNLKSSSSSSKQSSSVNAQSPSSGQLRTNHQFPFSPTLYNLTQLGGIGLNLAATNGNNQAHQIGSSLMMSDLGFLHGRNTSTPMTGNIHENNNNNNNENNLMASVGSLSPFALFDPTTGLYAFQNDGNIGNNVGISGSSTSMVDSRVYQTPPVKMEEQPNLANLSRPVSGLTSPGNQTNQYFWPGSDFSGPSNDLL* |
| AT1G21340 | AtDOF1.2 | MLPYIGHNSYQQHQFPLPEMEIPEKWKLSYEQEAITAPACPRCASSNTKFCYYNNYSLSQPRYFCKGCRRYWTKGGSLRNIPVGGGCRKRSRSRQNSHKRFGRNENRPDGLINQDDGFQSSPPGSDIDLAAVFAQYVTDRSPSSTDNTTGSDQDSPITTTTHALESLSWDICQETDVDLGFYGEFNNLTQKTKEDQEVFGQFLQEDREEIFEFQGLLDDKEIQEILECSFSEEPDQLVSQGSFMINGDNWSSTDLTRFGI* |
| AT1G26790 | AtDOF1.3 | MSSKIGMSQVRDTPVKLFGWTITPVSHDPYSSSSHVLPDSSSSSSSSSLSLRPHMMNNQSVTDNTSLKLSSNLNNESKETSENSDDQHSEITTITSEEEKTTELKKPDKILPCPRCNSADTKFCYYNNYNVNQPRHFCRKCQRYWTAGGSMRIVPVGSGRRKNKGWVSSDQYLHITSEDTDNYNSSSTKILSFESSDSLVTERPKHQSNEVKINAEPVSQEPNNFQGLLPPQASPVSPPWPYQYPPNPSFYHMPVYWGCAIPVWSTLDTSTCLGKRTRDETSHETVKESKNAFERTSLLLESQSIKNETSMATNNHVWYPVPMTREKTQEFSFFSNGAETKSSNNRFVPETYLNLQANPAAMARSMNFRESI* |
| At1g28310 | AtDOF1.4 | MFGNCDQNKKMPIISSPNTNPLASMQSKNMIVASSHQQQQQQQPQQPQPQLKCPRCDSSNTKFCYYNNYSLSQPRHFCKACKRYWTRGGTLRNVPVGGSYRKNKRVKRPSTATTTTASTVSTTNSSSPNNPHQISHFSSMNHHPLFYGLSDHMSSCNNNLPMIPSRFSDSSKTCSSSGLESEFLSSGFSSLSALGLGLPHQMSHDHTINGSFINNSTTNKPFLLSGLFGSSMSSSSTLLQHPHKPMNNGGDMLGQSHLQTLASLQDLHVGGNNEDMKYKEGKLDQISGNINGFMSSSSSLDPSNYNNMWNNASVVNGAWLDPTNNNVGSSLTSLI* |
| AT1G29160 | COG1 | MATQDSQGIKLFGKTITFNANITQTIKKEEQQQQQQPELQATTAVRSPSSDLTAEKRPDKIIPCPRCKSMETKFCYFNNYNVNQPRHFCKGCQRYWTAGGALRNVPVGAGRRKSKPPGRVGGFAELLGAATGAVDQVELDALLVEEWRAATASHGGFRHDFPVKRLRCYTDGQSC* |
| At1g47650 | AtDOF1.6 | MPSEPNQTRPTRVQPSTAAYPPPNLAEPLPCPRCNSTTTKFCYYNNYNLAQPRYYCKSCRRYWTQGGTLRDVPVGGGTRRSSSKRHRSFSTTATSSSSSSSVITTTTQEPATTEASQTKVTNLISGHGSFASLLGLGSGNGGLDYGFGYGYGLEEMSIGYLGDSSVGEIPVVDGCGGDTWQIGEIEGKSGGDSLIWPGLEISMQTNDVK* |
| AT1G51700 | ADOF1 | MQDLTSAAAYYHQSMMMTTAKQNQPELPEQEQLKCPRCDSPNTKFCYYNNYNLSQPRHFCKNCRRYWTKGGALRNIPVGGGTRKSNKRSGSSPSSNLKNQTVAEKPDHHGSGSEEKEERVSGQEMNPTRMLYGLPVGDPNGASFSSLLASNMQMGGLVYESGSRWLPGMDLGLGSVRRSDDTWTDLAMNRMEKN* |
| AT1G64620 | AtDOF1.8 | MDTAKWPQEFVVKPMNEIVTNTCLKQQSNPPSPATPVERKARPEKDQALNCPRCNSLNTKFCYYNNYSLTQPRYFCKDCRRYWTAGGSLRNIPVGGGVRKNKRSSSNSSSSSPSSSSSSKKPLFANNNTPTPPLPHLNPKIGEAAATKVQDLTFSQGFGNAHEVKDLNLAFSQGFGIGHNHHSSIPEFLQVVPSSSMKNNPLVSTSSSLELLGISSSSASSNSRPAFMSYPNVHDSSVYTASGFGLSYPQFQEFMRPALGFSLDGGDPLRQEEGSSGTNNGRPLLPFESLLKLPVSSSSTNSGGNGNLKENNDEHSDHEHEKEEGEADQSVGFWSGMLSAGASAAASGGSWQ* |
| At1g69570 | AtDOF1.10 | MSKSRDTEIKLFGRTITSLLDVNCYDPSSLSPVHDVSSDPSKEDSSSSSSSCSPTIGPIRVPVKKSEQESNKFKDPYILSDLNEPPKAVSEISSPRSSKNNCDQQSEITTTTTTSTTSGEKSTALKKPDKLIPCPRCESANTKFCYYNNYNVNQPRYFCRNCQRYWTAGGSMRNVPVGSGRRKNKGWPSSNHYLQVTSEDCDNNNSGTILSFGSSESSVTETGKHQSGDTAKISADSVSQENKSYQGFLPPQVMLPNNSSPWPYQWSPTGPNASFYPVPFYWGCTVPIYPTSETSSCLGKRSRDQTEGRINDTNTTITTTRARLVSESLRMNIEASKSAVWSKLPTKPEKKTQGFSLFNGFDTKGNSNRSSLVSETSHSLQANPAAMSRAMNFRESMQQ* |
| AT2G06010 | ORG4 | MEPPSSRPEEPPSWEDLYKINLMPSELFLKFRKELQGLRVGVNLELYNEPTNDYHAKLVLKPLCPERKWKFIYEPLHQEVRVLSKKIPVTRFLNLQVGVGHNFQMNAIGWKWKLTSCLGGDGVSRIRNKTTLGLSPGIDFRFGWRADFVLPEVTGALGTEEPLFNMSSGRLEASLDRVEAIVTHSDYL* |
| AT2G18915 | ADO2 | MQNQMEWDSDSDLSGGDEVAEDGWFGGDNGAIPFPVGSLPGTAPCGFVVSDALEPDNPIIYVNTVFEIVTGYRAEEVIGRNCRFLQCRGPFTKRRHPMVDSTIVAKMRQCLENGIEFQGELLNFRKDGSPLMNKLRLVPIREEDEITHFIGVLLFTDAKIDLGPSPDLSAKEIPRISRSFTSALPIGERNVSRGLCGIFELSDEVIAIKILSQLTPGDIASVGCVCRRLNELTKNDDVWRMVCQNTWGTEATRVLESVPGAKRIGWVRLAREFTTHEATAWRKFSVGGTVEPSRCNFSACAVGNRIVIFGGEGVNMQPMNDTFVLDLGSSSPEWKSVLVSSPPPGRWGHTLSCVNGSRLVVFGGYGSHGLLNDVFLLDLDADPPSWREVSGLAPPIPRSWHSSCTLDGTKLIVSGGCADSGALLSDTFLLDLSMDIPAWREIPVPWTPPSRLGHTLTVYGDRKILMFGGLAKNGTLRFRSNDVYTMDLSEDEPSWRPVIGYGSSLPGGMAAPPPRLDHVAISLPGGRILIFGGSVAGLDSASQLYLLDPNEEKPAWRILNVQGGPPRFAWGHTTCVVGGTRLVVLGGQTGEEWMLNEAHELLLATSTTAST* |
| At2g28510 | AtDOF2.1 | MDPEQEISNETLETILVSSTKGSNNNNKKMEEEMKKKVSRGELGGEAQNCPRCESPNTKFCYYNNYSLSQPRYFCKSCRRYWTKGGTLRNVPVGGGCRRNKRSSSSAFSKNNNNKSINFHTDPLQNPLITGMPPSSFGYDHSIDLNLAFATLQKHHLSSQATTPSFGFGGDLSIYGNSTNDVGIFGGQNGTYNNSLCYGFMSGNGNNNQNEIKMASTLGMSLEGNERKQENVNNNNNNSENPSKVFWGFPWQMTGDSAGVVPEIDPGRESWNGMVSSWNNGLLNTPLV* |
| At2g28810 | AtDof2.2 | MVFSSVSSFLDPPINWPQSANPNNHPHHHQLQENGSLVSGHHQVLSHHFPQNPNPNHHHVETAAATTVDPSSLNGQAAERARLAKNSQPPEGALKCPRCDSANTKFCYFNNYNLTQPRHFCKACRRYWTRGGALRNVPVGGGCRRNKKGKSGNSKSSSSSQNKQSTSMVNATSPTNTSNVQLQTNSQFPFLPTLQNLTQLGGIGLNLAAINGNNGGNGNTSSSFLNDLGFFHGGNTSGPVMGNNNENNLMTSLGSSSHFALFDRTMGLYNFPNEVNMGLSSIGATRVSQTAQVKMEDNHLGNISRPVSGLTSPGNQSNQYWTGQGLPGSSSNDHHHQHLM* |
| AT2G34140 | CDF4 | MATQDSQGIKLFGKTIAFNTRTIKNEEETHPPEQEATIAVRSSSSSDLTAEKRPDKIIACPRCKSMETKFCYFNNYNVNQPRHFCKGCHRYWTAGGALRNVPVGAGRRKSKPPGRVVVGMLGDGNGVRQVELINGLLVEEWQHAAAAAHGSFRHDFPMKRLRCYSDGQSC* |
| AT2G37590 | AtDOF2.4 | MVFSSIQAYLDSSNWQQAPPSNYNHDGTGASANGGHVLRPQLQPQQQPQQQPHPNGSGGGGGGGGGSIRAGSMVDRARQANVALPEAALKCPRCESTNTKFCYFNNYSLTQPRHFCKTCRRYWTRGGALRNVPVGGGCRRNRRTKSNSNNNNNSTATSNNTSFSSGNASTISTILSSHYGGNQESILSQILSPARLMNPTYNHLGDLTSNTKTDNNMSLLNYGGLSQDLRSIHMGASGGSLMSCVDEWRSASYHQQSSMGGGNLEDSSNPNPSANGFYSFESPRITSASISSALASQFSSVKVEDNPYKWVNVNGNCSSWNDLSAFGSSR* |
| AT2G46590 | DAG2 | MDATKWTQGFQEMMNVKPMEQIMIPNNNTHQPNTTSNARPNTILTSNGVSTAGATVSGVSNNNNNTAVVAERKARPQEKLNCPRCNSTNTKFCYYNNYSLTQPRYFCKGCRRYWTEGGSLRNVPVGGSSRKNKRSSSSSSSNILQTIPSSLPDLNPPILFSNQIHNKSKGSSQDLNLLSFPVMQDQHHHHVHMSQFLQMPKMEGNGNITHQQQPSSSSSVYGSSSSPVSALELLRTGVNVSSRSGINSSFMPSGSMMDSNTVLYTSSGFPTMVDYKPSNLSFSTDHQGLGHNSNNRSEALHSDHHQQGRVLFPFGDQMKELSSSITQEVDHDDNQQQKSHGNNNNNNNSSPNNGYWSGMFSTTGGGSSW* |
| AT3G21270 | ADOF2 | MQDPAAYYQTMMAKQQQQQQPQFAEQEQLKCPRCDSPNTKFCYYNNYNLSQPRHFCKSCRRYWTKGGALRNVPVGGGSRKNATKRSTSSSSSASSPSNSSQNKKTKNPDPDPDPRNSQKPDLDPTRMLYGFPIGDQDVKGMEIGGSFSSLLANNMQLGLGGGGIMLDGSGWDHPGMGLGLRRTEPGNNNNNPWTDLAMNRAEKN* |
| AT3G45610 | DOF6 | MDYSSMHQNVMGVSSCSTQDYQNQKKPLSATRPAPPEQSLRCPRCDSTNTKFCYYNNYSLSQPRYFCKSCRRYWTKGGILRNIPIGGAYRKHKRSSSATKSLRTTPEPTMTHDGKSFPTASFGYNNNNISNEQMELGLAYALLNKQPLGVSSHLGFGSSQSPMAMDGVYGTTSHQMENTGYAFGNGGGGMEQMATSDPNRVLWGFPWQMNMGGGSGHGHGHVDQIDSGREIWSSTVNYINTGALL* |
| AT3G47500 | CDF3 | MMMETRDPAIKLFGMKIPFPSVFESAVTVEDDEEDDWSGGDDKSPEKVTPELSDKNNNNCNDNSFNNSKPETLDKEEATSTDQIESSDTPEDNQQTTPDGKTLKKPTKILPCPRCKSMETKFCYYNNYNINQPRHFCKACQRYWTAGGTMRNVPVGAGRRKNKSSSSHYRHITISEALEAARLDPGLQANTRVLSFGLEAQQQHVAAPMTPVMKLQEDQKVSNGARNRFHGLADQRLVARVENGDDCSSGSSVTTSNNHSVDESRAQSGSVVEAQMNNNNNNNMNGYACIPGVPWPYTWNPAMPPPGFYPPPGYPMPFYPYWTIPMLPPHQSSSPISQKCSNTNSPTLGKHPRDEGSSKKDNETERKQKAGCVLVPKTLRIDDPNEAAKSSIWTTLGIKNEAMCKAGGMFKGFDHKTKMYNNDKAENSPVLSANPAALSRSHNFHEQI* |
| AT3G50410 | OBP1 | MPTSDSGEPRRIAMKPNGVTVPISDQQEQLPCPRCDSSNTKFCYYNNYNFSQPRHFCKACRRYWTHGGTLRDVPVGGGTRKSAKRSRTCSNSSSSSVSGVVSNSNGVPLQTTPVLFPQSSISNGVTHTVTESDGKGSALSLCGSFTSTLLNHNAAATATHGSGSVIGIGGFGIGLGSGFDDVSFGLGRAMWPFSTVGTATTTNVGSNGGHHAVPMPATWQFEGLESNAGGGFVSGEYFAWPDLSITTPGNSLK* |
| At3g52440 | AtDOF3.5 | MERAEALTSSFIWRPNANANAEITPSCPRCGSSNTKFCYYNNYSLTQPRYFCKGCRRYWTKGGSLRNVPVGGGCRKSRRPKSSSGNNTKTSLTANSGNPGGGSPSIDLALVYANFLNPKPDESILQENCDLATTDFLVDNPTGTSMDPSWSMDINDGHHDHYINPVEHIVEECGYNGLPPFPGEELLSLDTNGVWSDALLIGHNHVDVGVTPVQAVHEPVVHFADESNDSTNLLFGSWSPFDFTADG* |
| AT3G55370 | OBP3 | MVFSSLPVNQFDSQNWQQMISILVFFSTSRLFKKLFLVDKNLFSCLLQGLMYNVFLTGLIFSLQGNQHQLECVTTDQNPNNYLRQLSSPPTSQVAGSSQARVNSMVERARIAKVPLPEAALNCPRCDSTNTKFCYFNNYSLTQPRHFCKTCRRYWTRGGSLRNVPVGGGFRRNKRSKSRSKSTVVVSTDNTTSTSSLTSRPSYSNPSKFHSYGQIPEFNSNLPILPPLQSLGDYNSSNTGLDFGGTQISNMISGMSSSGGILDAWRIPPSQQAQQFPFLINTTGLVQSSNALYPLLEGGVSATQTRNVKAEENDQDRGRDGDGVNNLSRNFLGNININSGRNEEYTSWGGNSSWTGFTSNNSTGHLSF* |
| At3g61850 | DAG1 | MDATKWTQGFQEMINVKPMEQMISSTNNNTPQQQPTFIATNTRPNATASNGGSGGNTNNTATMETRKARPQEKVNCPRCNSTNTKFCYYNNYSLTQPRYFCKGCRRYWTEGGSLRNVPVGGSSRKNKRSSTPLASPSNPKLPDLNPPILFSSQIPNKSNKDLNLLSFPVMQDHHHHGMSHFFHMPKIENNNTSSSIYASSSPVSALELLRSNGVSSRGMNTFLPGQMMDSNSVLYSSLGFPTMPDYKQSNNNLSFSIDHHQGIGHNTINSNQRAQDNNDDMNGASRVLFPFSDMKELSSTTQEKSHGNNTYWNGMFSNTGGSSW* |
| AT4G00940 | ITD1 | MDHHQYHHHDQYQHQMMTSTNNNSYNTIVTTQPPPTTTTMDSTTATTMIMDDEKKLMTTMSTRPQEPRNCPRCNSSNTKFCYYNNYSLAQPRYLCKSCRRYWTEGGSLRNVPVGGGSRKNKKLPFPNSSTSSSTKNLPDLNPPFVFTSSASSSNPSKTHQNNNDLSLSFSSPMQDKRAQGHYGHFSEQVVTGGQNCLFQAPMGMIQFRQEYDHEHPKKNLGFSLDRNEEEIGNHDNFVVNEEGSKMMYPYGDHEDRQQHHHVRHDDGNKKREGGSSNELWSGIILGGDSGGPTW* |
| AT4G21030 | AtDOF4.2 | MNNLNVFTNEDNEMNVMPPPRVCPRCYSDQTRFSYFNNNKKSQPRYKCKNCCRCWTHGGVLRNIPVTGICDKSNLPKIDQSSVSQMILAEIQQGNHQPFKKFQENISVSVSSSSDVSIVGNHFDDLSELHGITNSTPIRSFTMDRLDFGEESFQQDLYDVGSNDLIGNPLINQSIGGYVDNHKDEHKLQFEYES* |
| AT4G21040 | AtDOF4.3 | MDNFNVVANEDNQVNDVKPPPPPPRVCARCDSDNTKFCYYNNYSEFQPRYFCKNCRRYWTHGGALRNVPIGGSSRAKRTRINQPSVAQMVSVGIQPGSHKPFFNVQENNDFVGSFGASSSSFVAAVGNRFSSLSHIHGGMVTNVHPTQTFRPNHRLAFHNGSFEQDYYDVGSDNLLVNQQVGGYVDNHNGYHMNQVDQYNWNQSFNNAMNMNYNNASTSGRMHPSHLEKGGP* |
| AT4G21050 | AtDOF4.4 | MDNLNVFANEDNQVNGLKRPPPSRVCPRCDSDNTKFCFYNNYSESQPRYFCKNCRRYWTHGGALRNIPVGGSCRKPKRLKVDQSSISEMVSVENQPINHQSFRQTQENNEFVRSFDASSSATVTAVPNHFGYLSELHGVTNLLPIQSFRTMDCLDFGDESFQQGYYDVGSNDLIDNPLINQSIGGYVDNLTSYCINQVEPKLQPRYEHES* |
| AT4G21080 | AtDOF4.5 | MDNLNVFANEDNQVNDVKPPPPPPRVCARCDSDNTKFCYYNNYCEFQPRYFCKNCRRYWTHGGALRNIPIGGSSRAKRARVNQPSVARMVSVETQRGNNQPFSNVQENVHLVGSFGASSSSSVGAVGNLFGSLYDIHGGMVTNLHPTRTVRPNHRLAFHDGSFEQDYYDVGSDNLLVNQQVGGYGYHMNPVDQFKWNQSFNNTMNMNYNNDSTSGSSRGSDMNVNHDNKKIRYRNSVIMHPCHLEKDGP* |
| At4g24060 | AtDOF4.6 | MDTAQWPQEIVVKPLEEIVTNTCPKPQPQPLQPQQPPSVGGERKARPEKDQAVNCPRCNSTNTKFCYYNNYSLTQPRYFCKGCRRYWTEGGSLRNIPVGGGSRKNKRSHSSSSDISNNHSDSTQPATKKHLSDHHHHLMSMSQQGLTGQNPKFLETTQQDLNLGFSPHGMIRTNFTDLIHNIGNNTNKSNNNNNPLIVSSCSAMATSSLDLIRNNSNNGNSSNSSFMGFPVHNQDPASGGFSMQDHYKPCNTNTTLLGFSLDHHHNNGFHGGFQGGEEGGEGGDDVNGRHLFPFEDLKLPVSSSSATINVDINEHQKRGSGSDAAATSGGYWTGMLSGGSWC* |
| At4g38000 | AtDof4.7 | MMTSSHQSNTTGFKPRRIKTTAKPPRQINNKEPSPATQPVLKCPRCDSVNTKFCYYNNYSLSQPRHYCKNCRRYWTRGGALRNVPIGGSTRNKNKPCSLQVISSPPLFSNGTSSASRELVRNHPSTAMMMMSSGGFSGYMFPLDPNFNLASSSIESLSSFNQDLHQKLQQQRLVTSMFLQDSLPVNEKTVMFQNVELIPPSTVTTDWVFDRFATGGGATSGNHEDNDDGEGNLGNWFHNANNNALL* |
| At5g02460 | AtDof5.1 | MVFSSFPTYPDHSSNWQQQHQPITTTVGFTGNNINQQFLPHHPLPPQQQQTPPQLHHNNGNGGVAVPGGPGGLIRPGSMAERARLANIPLPETALKCPRCDSTNTKFCYFNNYSLTQPRHFCKACRRYWTRGGALRSVPVGGGCRRNKRTKNSSGGGGGSTSSGNSKSQDSATSNDQYHHRAMANNQMGPPSSSSSLSSLLSSYNAGLIPGHDHNSNNNNILGLGSSLPPLKLMPPLDFTDNFTLQYGAVSAPSYHIGGGSSGGAAALLNGFDQWRFPATNQLPLGGLDPFDQQHQMEQQNPGYGLVTGSGQYRPKNIFHNLISSSSSASSAMVTATASQLASVKMEDSNNQLNLSRQLFGDEQQLWNIHGAAAASTAAATSSWSEVSNNFSSSSTSNI* |
| [AT5G23040](https://www.arabidopsis.org/servlets/TairObject?id=135215&type=locus) | CPP1 | MSSSLLLSGSTVSSSFIAPSKPSLVRNSSKTSLLPFRNVSRSFKTVKCTVDSSYGGNVPTFPRTRVWDPYKRLGVSPYASEEEIWASRNFLLQQYAGHERSEESIEGAFEKLLMSSFIRRKKTKINLKSKLKKKVEESPPWLKALLDFVEMPPMDTIFRRLFLFAFMGGWSIMNSAEGGPAFQVAVSLAACVYFLNEKTKSLGRACLIGIGALVAGWFCGSLIIPMIPTFLIQPTWTLELLTSLVAYVFLFLSCTFLK* |
| AT5G39660 | CDF2 | MADPAIKLFGKTIPLPELGVVDSSSSYTGFLTETQIPVRLSDSCTGDDDDEEMGDSGLGREEGDDVGDGGGESETDKKEEKDSECQEESLRNESNDVTTTTSGITEKTETTKAAKTNEESGGTACSQEGKLKKPDKILPCPRCNSMETKFCYYNNYNVNQPRHFCKKCQRYWTAGGTMRNVPVGAGRRKNKSPASHYNRHVSITSAEAMQKVARTDLQHPNGANLLTFGSDSVLCESMASGLNLVEKSLLKTQTVLQEPNEGLKITVPLNQTNEEAGTVSPLPKVPCFPGPPPTWPYAWNGVSWTILPFYPPPAYWSCPGVSPGAWNSFTWMPQPNSPSGSNPNSPTLGKHSRDENAAEPGTAFDETESLGREKSKPERCLWVPKTLRIDDPEEAAKSSIWETLGIKKDENADTFGAFRSSTKEKSSLSEGRLPGRRPELQANPAALSRSANFHESS* |
| AT5G60200 | TMO6 | MDHLLQHQDVFGNYNKAREAMGLSYSSNPTPLDNDQKKPSPATAVTRPQPPELALRCPRCDSTNTKFCYYNNYSLTQPRYFCKSCRRYWTKGGTLRNIPVGGGCRKNKRSTSSAARSLRTTPEPASHDGKVFSAAGFNGYSNNEHIDLSLAFALLNKQHPGSSSQLGFHSELGSSHQSDMEGMFGTSQQKENATYAFGNGSSGLGDPSRVLWGFPWQMNGESFGMMNIGGGGGHVDQIDSGREMWTNMNYINSGALM* |
| At5g60850 | OBP4 | MQDIHDFSMNGVGGGGGGGGRFFGGGIGGGGGGDRRMRAHQNNILNHHQSLKCPRCNSLNTKFCYYNNYNLSQPRHFCKNCRRYWTKGGVLRNVPVGGGCRKAKRSKTKQVPSSSSADKPTTTQDDHHVEEKSSTGSHSSSESSSLTASNSTTVAAVSVTAAAEVASSVIPGFDMPNMKIYGNGIEWSTLLGQGSSAGGVFSEIGGFPAVSAIETTPFGFGGKFVNQDDHLKLEGETVQQQQFGDRTAQVEFQGRSSDPNMGFEPLDWGSGGGDQTLFDLTSTVDHAYWSQSQWTSSDQDQSGLYLP* |
| AT5G23040 | CDF1 | MLETKDPAIKLFGMKIPFPTVLEVADEEEEKNQNKTLTDQSEKDKTLKKPTKILPCPRCNSMETKFCYYNNYNVNQPRHFCKACQRYWTSGGTMRSVPIGAGRRKNKNNSPTSHYHHVTISETNGPVLSFSLGDDQKVSSNRFGNQKLVARIENNDERSNNNTSNGLNCFPGVSWPYTWNPAFYPVYPYWSMPVLSSPVSSSPTSTLGKHSRDEDETVKQKQRNGSVLVPKTLRIDDPNEAAKSSIWTTLGIKNEVMFNGFGSKKEVKLSNKEETETSLVLCANPAALSRSINFHEQM* |
| AT5G62940 | HCA2 | MGLTSLQVCMDSDWLQESESSGGSMLDSSTNSPSAADILAACSTRPQASAVAVAAAALMDGGRRLRPPHDHPQKCPRCESTHTKFCYYNNYSLSQPRYFCKTCRRYWTKGGTLRNIPVGGGCRKNKKPSSSNSSSSTSSGKKPSNIVTANTSDLMALAHSHQNYQHSPLGFSHFGGMMGSYSTPEHGNVGFLESKYGGLLSQSPRPIDFLDSKFDLMGVNNDNLVMVNHGSNGDHHHHHNHHMGLNHGVGLNNNNNNGGFNGISTGGNGNGGGLMDISTCQRLMLSNYDHHHYNHQEDHQRVATIMDVKPNPKLLSLDWQQDQCYSNGGGSGGAGKSDGGGYGNGGYINGLGSSWNGLMNGYGTSTKTNSLV* |
| AT5G65590 | SCAP1 | MSSHTNLPSPKPVPKPDHRISGTSQTKKPPSSSVAQDQQNLKCPRCNSPNTKFCYYNNYSLSQPRHFCKSCRRYWTRGGALRNVPIGGGCRKTKKSIKPNSSMNTLPSSSSSQRFFSSIMEDSSKFFPPPTTMDFQLAGLSLNKMNDLQLLNNQEVLDLRPMMSSGRENTPVDVGSGLSLMGFGDFNNNHSPTGFTTAGASDGNLASSIETLSCLNQDLHWRLQQQRMAMLFGNSKEETVVVERPQPILYRNLEIVNSSSPSSPTKKGDNQTEWYFGNNSDNEGVISNNANTGGGGSEWNNGIQAWTDLNHYNALP* |
| AT5G66940 | AtDof5.8 | MPSEFSESRRVPKIPHGQGGSVAIPTDQQEQLSCPRCESTNTKFCYYNNYNFSQPRHFCKSCRRYWTHGGTLRDIPVGGVSRKSSKRSRTYSSAATTSVVGSRNFPLQATPVLFPQSSSNGGITTAKGSASSFYGGFSSLINYNAAVSRNGPGGGFNGPDAFGLGLGHGSYYEDVRYGQGITVWPFSSGATDAATTTSHIAQIPATWQFEGQESKVGFVSGDYVA* |
