# Supplementary figures and images for: Genome-wide analysis of BpDof genes and the tolerance to drought stress in birch (Betula platyphylla)
Source: PeerJ. 2021 Aug 24;9:e11938. doi: 10.7717/peerj.11938 (PMC8395574; doi:10.7717/peerj.11938)

**Table S5. All BpDof proteins localization prediction.**
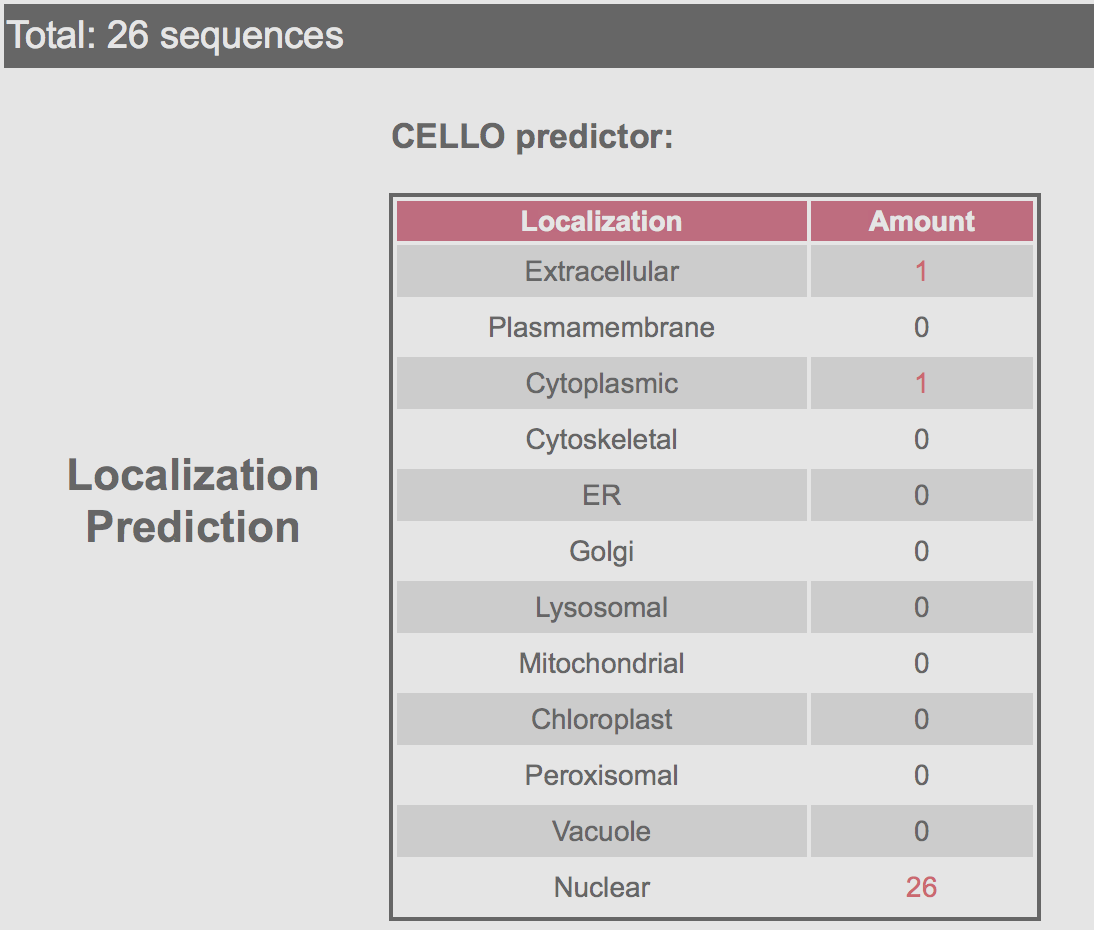

Supplement: Supplemental Information 5 [file peerj-09-11938-s005.docx]
