# Supplementary material for: Genome-wide analysis of BpDof genes and the tolerance to drought stress in birch (Betula platyphylla)
Source: PeerJ. 2021 Aug 24;9:e11938. doi: 10.7717/peerj.11938 (PMC8395574; doi:10.7717/peerj.11938)

**Table S6. All BpDof proteins transmembrane domain analyses.**

| Locus ID | Number of predicted TMHs | Exp number of AAs in TMHs | Exp number, first 60 AAs | Total prob of N-in |
| --- | --- | --- | --- | --- |
| BPChr06G16490 | 0 | 0.00086 | 0 | 0.00333 |
| BPChr12G11401 | 0 | 0.06735 | 0 | 0.03807 |
| BPChr11G05806 | 0 | 0.00131 | 0.00023 | 0.00158 |
| BPChr12G08354 | 0 | 0 | 0 | 0.02339 |
| BPChr14G09159 | 0 | 0.00072 | 0.10016 | 0 |
| BPChr06G09621 | 0 | 0 | 0 | 0.0038 |
| BPChr14G12625 | 0 | 0.00393 | 0 | 0.00335 |
| BPChr12G29175 | 0 | 0.00237 | 0.00092 | 0.01404 |
| BPChr06G29469 | 0 | 0.00046 | 0 | 0.01695 |
| BPChr02G19918 | 0 | 0.00188 | 0 | 0.06505 |
| BPChr10G04282 | 0 | 0 | 0 | 0.01802 |
| BPChr06G19208 | 0 | 0.00238 | 0 | 0.04032 |
| BPChr03G28866 | 0 | 0.00037 | 0 | 0.03645 |
| BPChr04G00494 | 0 | 0.00499 | 0.00183 | 0.00103 |
| BPChr04G23864 | 0 | 0.00268 | 0 | 0.01185 |
| BPChr06G02126 | 0 | 1.06509 | 0 | 0.12438 |
| BPChr07G09798 | 0 | 0.00118 | 0.00069 | 0.10213 |
| BPChr07G18918 | 0 | 0.06878 | 0 | 0.0287 |
| BPChr07G18939 | 0 | 0.00678 | 0 | 0.0092 |
| BPChr08G01518 | 0 | 0.00309 | 0.00018 | 0.08748 |
| BPChr08G17028 | 0 | 0.00089 | 0 | 0.00291 |
| BPChr11G09292 | 0 | 0.00042 | 0 | 0.00437 |
| BPChr11G10185 | 0 | 0.004 | 0 | 0.04955 |
| BPChr12G29204 | 0 | 0.00452 | 0.00018 | 0.05301 |
| BPChr13G02551 | 0 | 0.00679 | 0 | 0.00272 |
| BPChr14G05515 | 0 | 0.20956 | 0.00018 | 0.01146 |


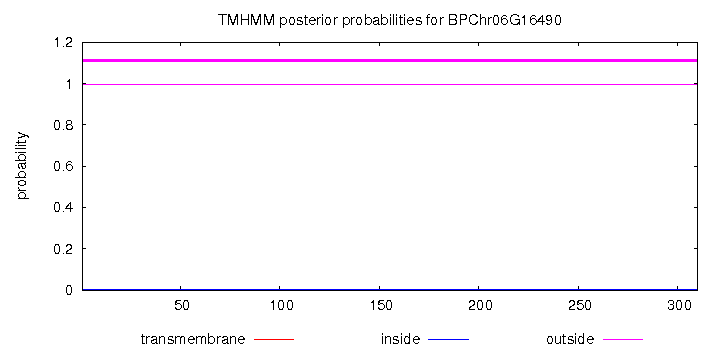

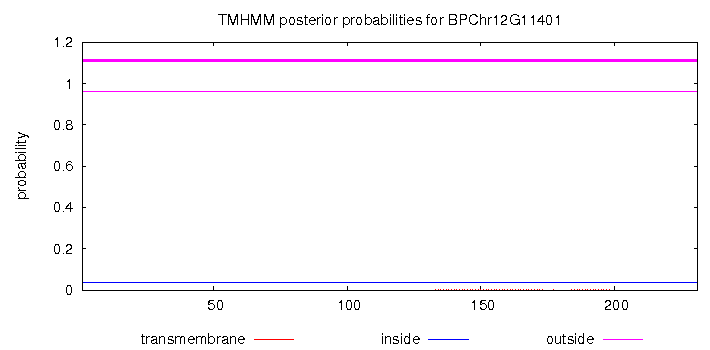

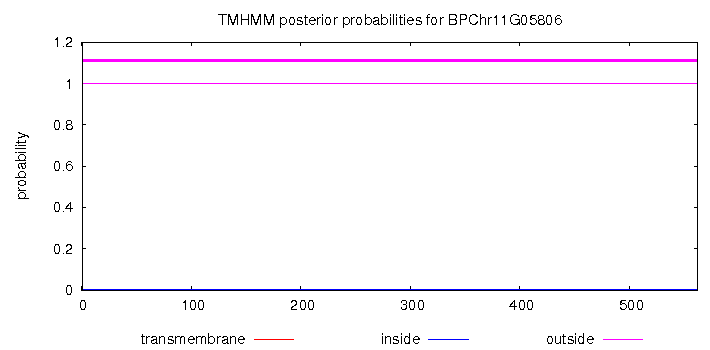

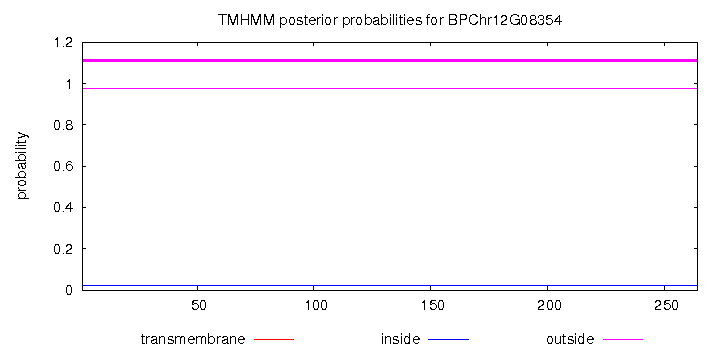

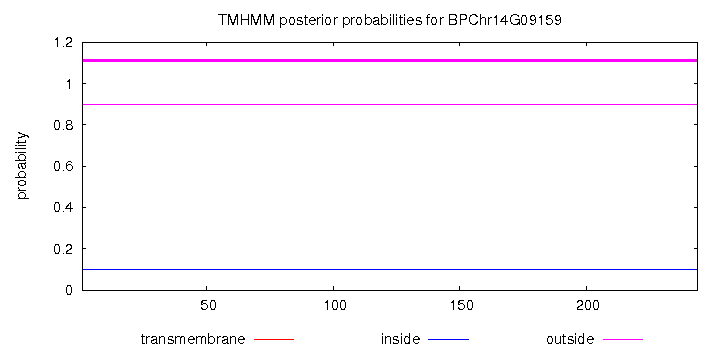

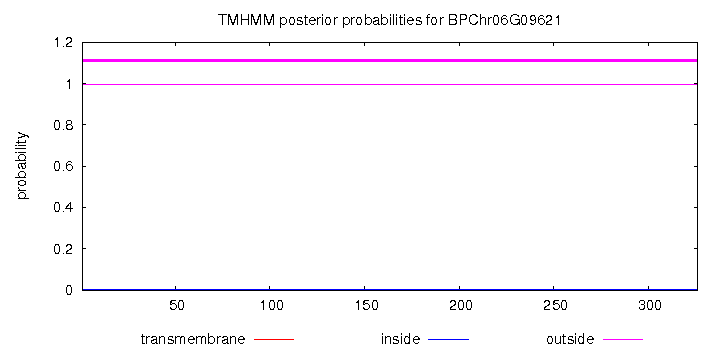


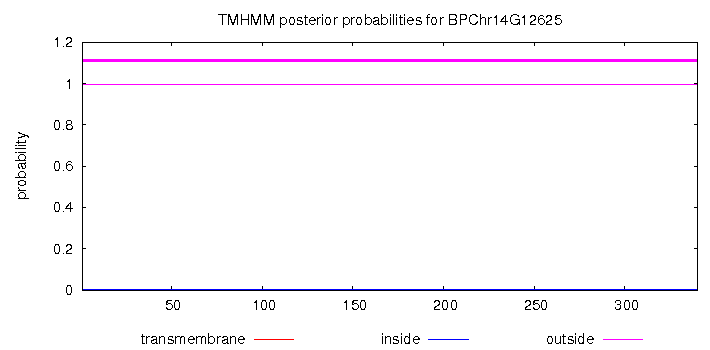

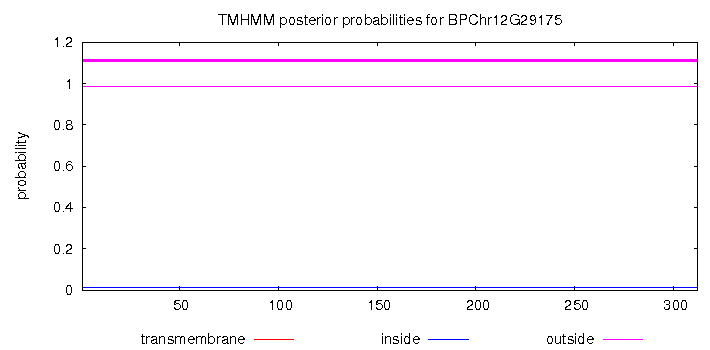

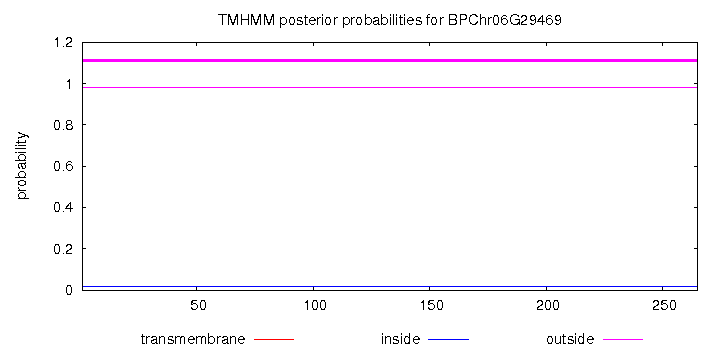

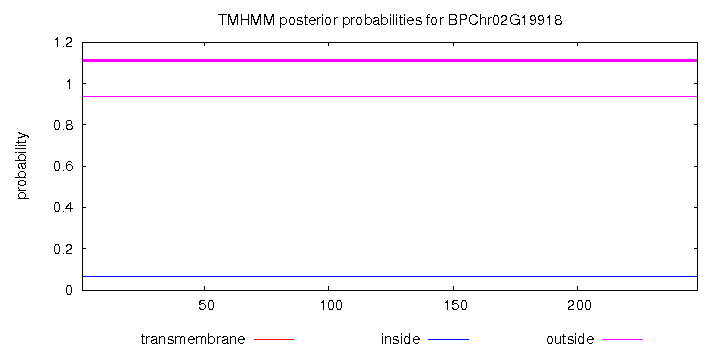

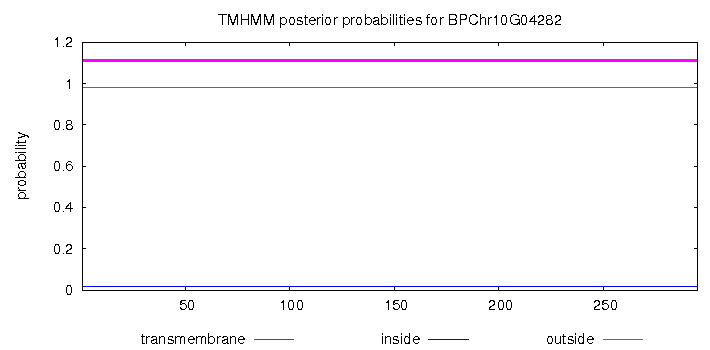

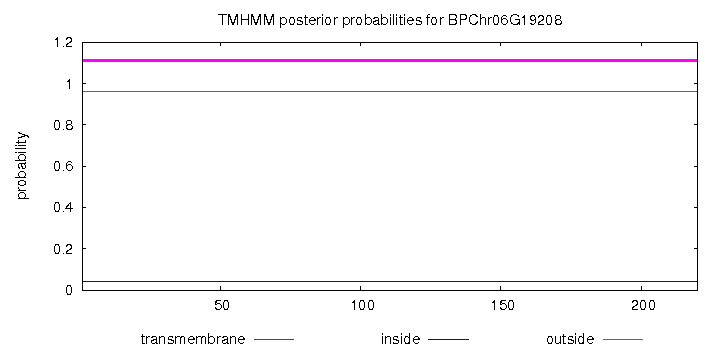

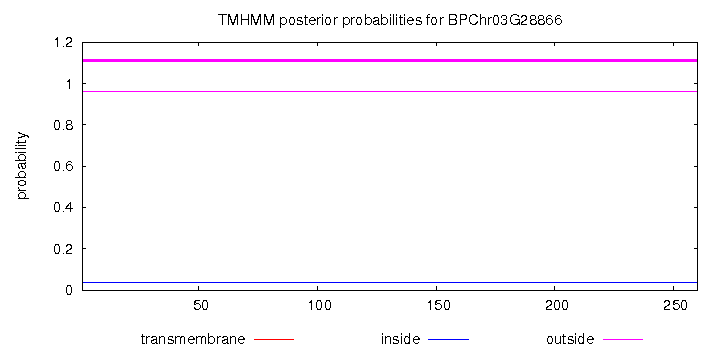

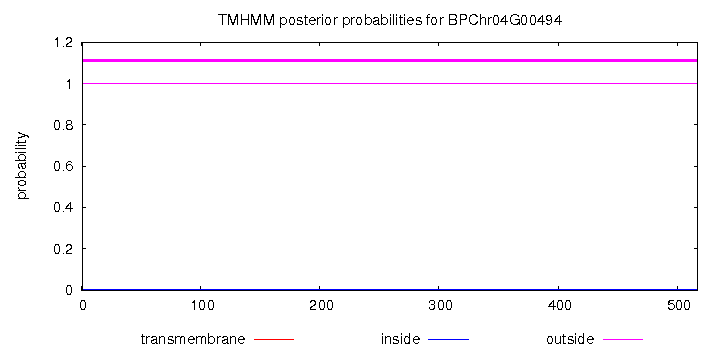

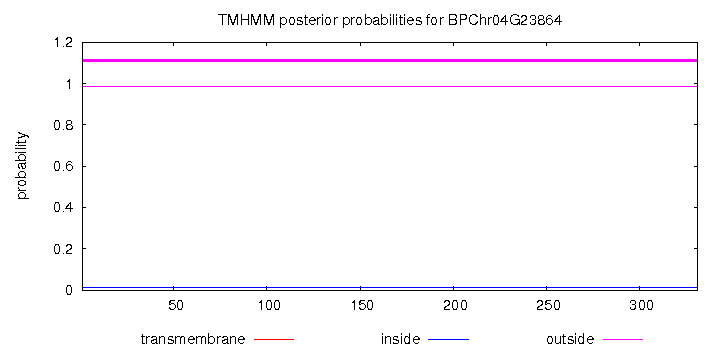

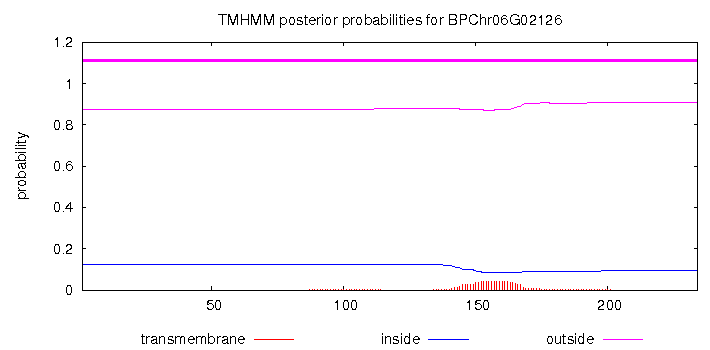

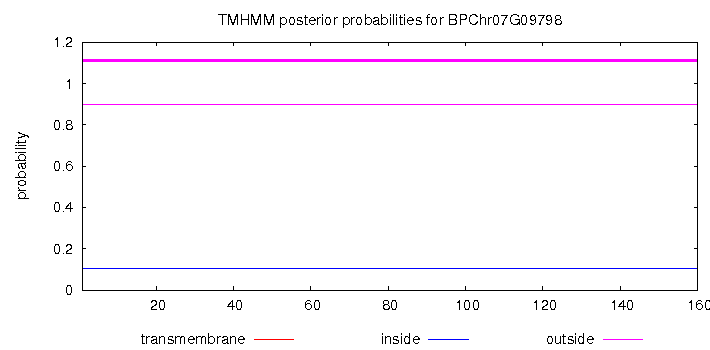

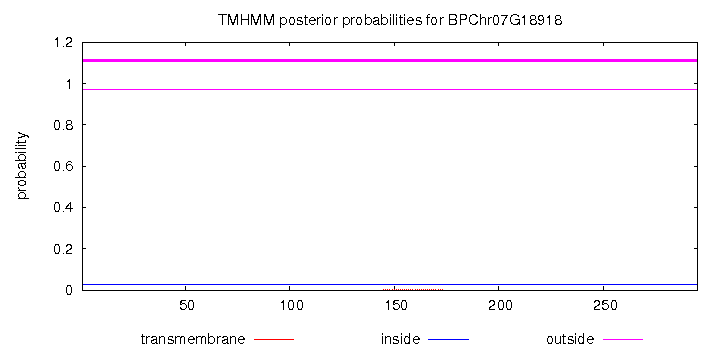

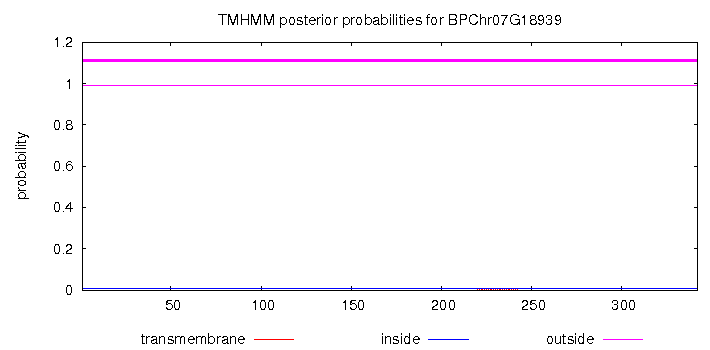

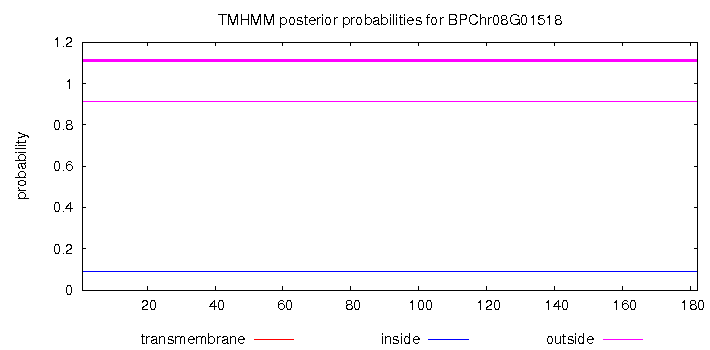

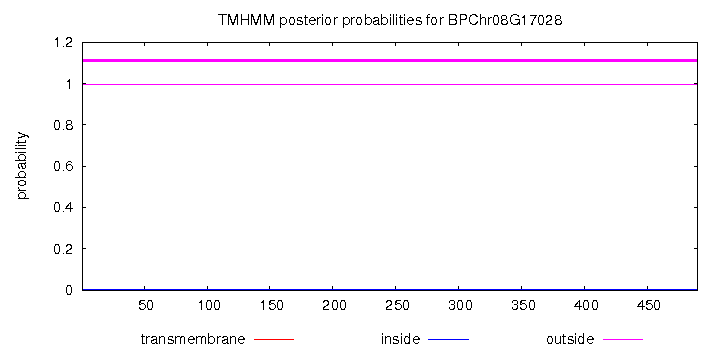

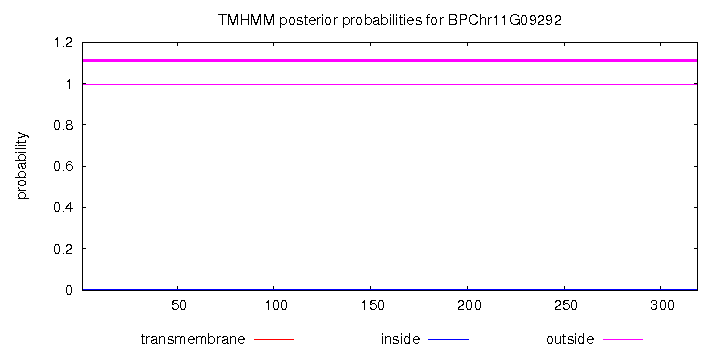

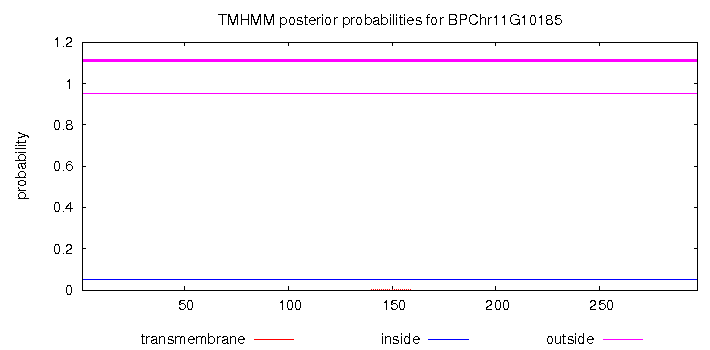

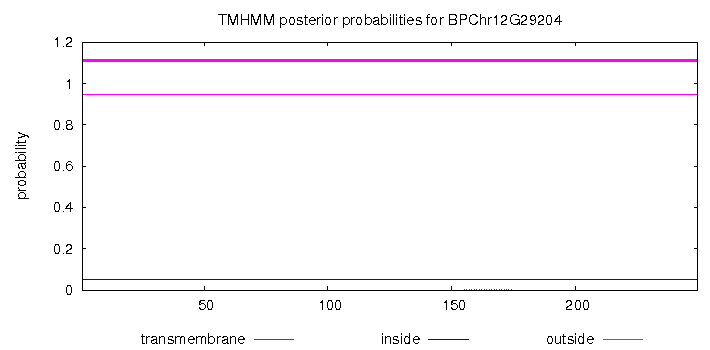

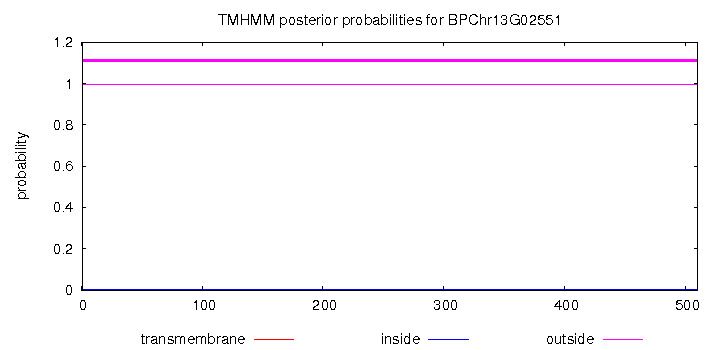

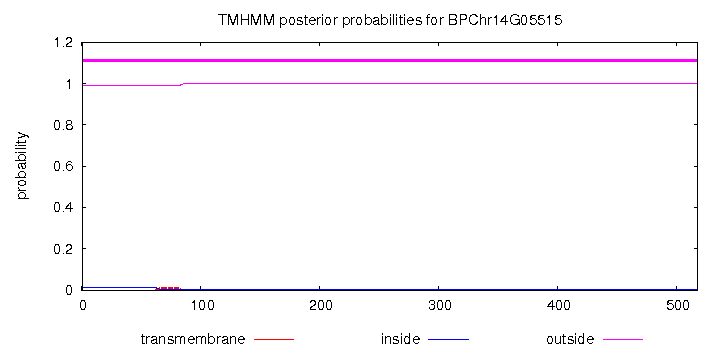

Supplement: Supplemental Information 6 [file peerj-09-11938-s006.docx]
