# Supplementary material for: Genome-wide analysis of BpDof genes and the tolerance to drought stress in birch (Betula platyphylla)
Source: PeerJ. 2021 Aug 24;9:e11938. doi: 10.7717/peerj.11938 (PMC8395574; doi:10.7717/peerj.11938)
Supplement: Supplemental Information 7 [file peerj-09-11938-s007.docx]

**Table S5.** Gene codes and amino acid sequences of *AtDof* TFs and *PtDof* TFs for alignment.

| Gene code. | amino acid sequences |
| --- | --- |
| AT1G07640 | MAFPSNWSQPTNSNHQHHLQHQLNENGSIISGHGLVLSHQLPPLQANPNPNHHHVATSAGLPSRMGGSMAERARQANIPPLAGPLKCPRCDSSNTKFCYYNNYNLTQPRHFCKGCRRYWTQGGALRNVPVGGGCRRNNKKGKNGNLKSSSSSSKQSSSVNAQSPSSGQLRTNHQFPFSPTLYNLTQLGGIGLNLAATNGNNQAHQIGSSLMMSDLGFLHGRNTSTPMTGNIHENNNNNNNENNLMASVGSLSPFALFDPTTGLYAFQNDGNIGNNVGISGSSTSMVDSRVYQTPPVKMEEQPNLANLSRPVSGLTSPGNQTNQYFWPGSDFSGPSNDLL* |
| AT3G55370 | MVFSSLPVNQFDSQNWQQMISILVFFSTSRLFKKLFLVDKNLFSCLLQGLMYNVFLTGLIFSLQGNQHQLECVTTDQNPNNYLRQLSSPPTSQVAGSSQARVNSMVERARIAKVPLPEAALNCPRCDSTNTKFCYFNNYSLTQPRHFCKTCRRYWTRGGSLRNVPVGGGFRRNKRSKSRSKSTVVVSTDNTTSTSSLTSRPSYSNPSKFHSYGQIPEFNSNLPILPPLQSLGDYNSSNTGLDFGGTQISNMISGMSSSGGILDAWRIPPSQQAQQFPFLINTTGLVQSSNALYPLLEGGVSATQTRNVKAEENDQDRGRDGDGVNNLSRNFLGNININSGRNEEYTSWGGNSSWTGFTSNNSTGHLSF* |
| Potri.015G009300 | MDTSTQWPQGIGVVKPVEGPDMLERRARPQKEQALNCPRCTSTNTKFCYYNNYSLSQPRYFCKTCRRYWTEGGSLRNVPVGGGSRKNKRSSSNPSSSAAAASEKKFPLDLTQPNFHQSATDQNPKIHQGPDLNLAYPPSHISAMELLKSSGMNPRGFSAFMSIPAASDSNNMFSTGFPLQEFPSTQNFSLEGFESGYSNIQAVHETGSSARLLFPIEDLKQQVPSNSEFERINARGQGDGAPGYWNGMLGGESW* |
| Potri.002G070700 | MYSASDQMMFQCPPRPFPMERKWKSNIEVAPNCPRCASPNTKFCYYNNYSLSQPRYFCKGCRRYWTKGGSLRNVPAGGGCRKYRRARSSKISQNERAAVSLDYSRANETLACSSNKDSVAQQDGANGSDIDLAAVFSKFLNQDLSYGPEFIGEELRDEGSELVNISNSSTPISDSYQNDPMMESLKRSDLTQESNLLEGRSQVLVGEKQQFEEDQRFQELIESQDMNAFGLQNLLIDEIVQDALWSDDATLPHVPNWQPMVQLQDFDSFSVDDRLKISANFTSDDNWSSFDLSGFEVFPRP* |
